# Proton-Transfer Dynamics Regulates CO_2_ Electroreduction Products via Hydrogen Coverage

**DOI:** 10.1021/acscentsci.4c01534

**Published:** 2024-11-28

**Authors:** Qun Fan, Tiantian Xiao, Hai Liu, Tianxiang Yan, Jianlong Lin, Siyu Kuang, Haoyuan Chi, Thomas J. Meyer, Sheng Zhang, Xinbin Ma

**Affiliations:** † Key Laboratory for Green Chemical Technology of Ministry of Education, Collaborative Innovation Centre of Chemical Science and Engineering, School of Chemical Engineering and Technology, Tianjin University, Tianjin 300072, China; ‡ Haihe Laboratory of Sustainable Chemical Transformations, Tianjin 300192, China; § Department of Chemistry, University of North Carolina at Chapel Hill, Chapel Hill, North Carolina 27599, United States

## Abstract

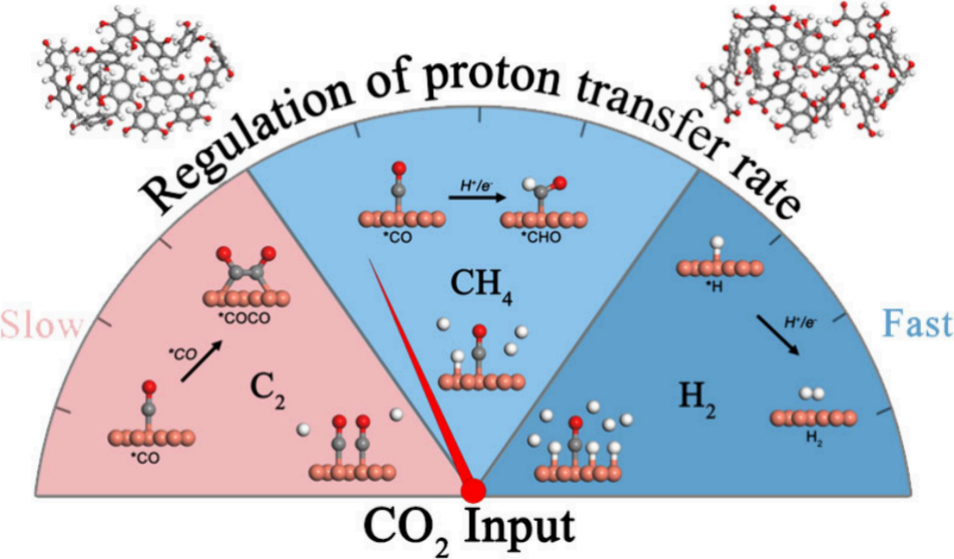

Electrochemical conversion of CO_2_ to hydrocarbons is a promising approach to carbon neutrality and energy storage. The formation of reaction intermediates involves crucial steps of proton transfer, making it essential to understand the role of protons in the electrochemical process to control the product selectivity and elucidate the underlying catalytic reaction mechanism of the CO_2_ electrochemical reduction (CO_2_RR). In this work, we proposed a strategy to regulate product selectivities by tuning local proton transport rates through a surface resin layer over cuprous oxides. We systematically studied the influence of proton transfer rates on product selectivities by regulating the polymerization degree of resorcinol-formaldehyde resin (RF). The production of C_2_ compounds and CH_4_ could be switched through an RF coating with the maximum CH_4_ Faradaic efficiency of 51% achieved at current densities close to the amperage level. Both experimental and theoretical calculation results suggest that the resin layer can subtly alter proton transfer rates during the electrochemical process, thereby influencing the hydrogen coverage on catalytic sites and ultimately guiding the overall electrochemical performance toward product selectivity.

## Abstract 2

Proton transfer rates are regulated through a surface resin layer to efficiently tune different product selectivities over copper catalysts during electrochemical CO_2_ reduction.

## Introduction

The electrochemical CO_2_ reduction reaction (CO_2_RR) has emerged as a promising strategy to reduce CO_2_ emissions and store intermittent renewable electricity^1−3^ in hydrocarbon products through multiple proton–electron transfer processes.^4^ However, significant challenges and controversies persist in producing hydrocarbon products with high selectivity and activity and understanding their associated catalytic mechanisms.^5^ Thus, it is crucial to develop an approach to achieving the efficient and selective production of hydrocarbons.

The regulation of local reaction environments such as the local pH, the choice of cation and anion species and their concentrations, and local reactant/intermediate concentrations has been demonstrated to largely influence the activity and selectivity of CO_2_RR products.^6−9^ Many of those efforts have focused on increasing the local concentration of CO_2_, such as by manipulating the local electric field,^10^ customizing the hydrophobicity of the catalyst,^11^ and incorporating organic additives.^12^ Protons are another key reactant for the CO_2_RR, which could affect the key intermediate by the protonation step. The previous works have reported that the selectivity of C_2+_ products over copper has been improved by enhancing water activation to supply more protons,^13,14^ while enhanced proton transfer was also reported to promote the generation of C_1_ products.^15,16^ There remains a lack of understanding regarding the mechanism underlying the relationship between proton availability and product selectivity.

It is challenging to precisely tune proton transport rates because of the complex chemical equilibria among CO_2_, hydroxide, bicarbonate, and carbonate.^17^ In addition, the local pH near the reaction interface can be significantly different from the bulk pH in the electrolyte, particularly at high current densities, which can reach up to 6 pH units higher.^18^ To this end, herein we present a facile interfacial engineering strategy to tune proton transfer rates. The Cu_2_O catalyst surface was coated with a resorcinol-formaldehyde resin (RF) layer as a proton shuttle to tune its transfer rate locally. *In situ* attenuated total reflection Fourier transform infrared spectroscopy (ATR-FTIR) was used to depict the proton migration path. Proton transfer rates could be adjusted through modulation of the resin layer, which reveals a relationship between product selectivities and proton transport rates. Density functional theory (DFT) calculations exhibited proton transfer-facilitated hydrogen coverage and thus regulated product selectivity. Overall, our interfacial engineering strategy for proton transport regulation provides insights into the role of protons in CO_2_RR kinetics.

## Results and Discussions

### Structure and Properties of RF-Coated Cu_2_O Nanocubes

**Figure 1:**
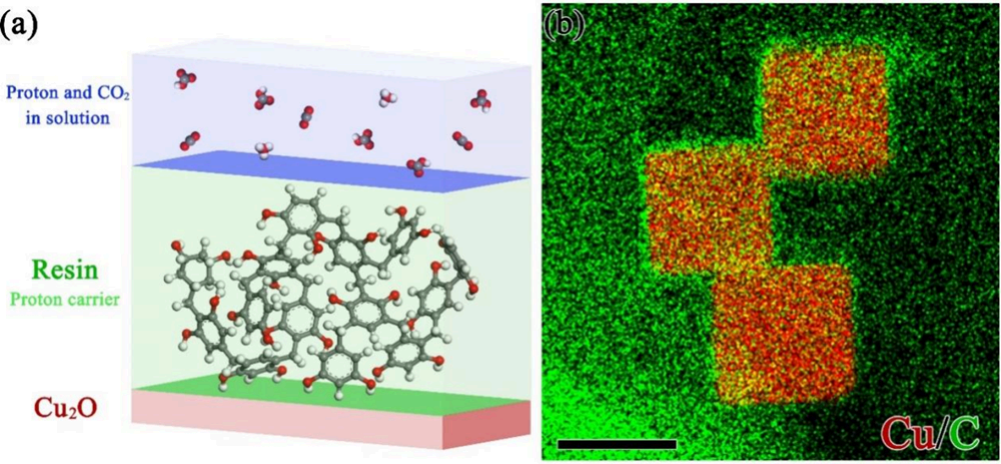
(a) Schematic of resins that controls the thermodynamics and kinetics of protons. (b) Elemental mappings of Cu_2_O@RF. The scale bars in these pictures are 50 nm.

The resorcinol-formaldehyde (RF) resin was coated on the surface of Cu_2_O (Figure 1), and the fabrication process is depicted in Scheme S1. RF is a condensation polymer, including resorcinol and formaldehyde linked by −CH_2_– bridges (Scheme S2). As-prepared Cu_2_O nanocubes were uniform with a particle size of ca. 50 nm (Figure S1), which remained unchanged after RF coating. XRD patterns (Figure S2) showed diffraction peaks of Cu_2_O only (JCPDS no. 05-0667), which indicated that the RF coating does not affect the crystal structure of Cu_2_O. Figure S3a shows X-ray photoelectron spectra (XPS) of Cu_2_O and Cu_2_O@RF samples, which featured a Cu 2p^3/2^ peak at 932.4 eV. However, the small binding energy difference of 0.1 eV between Cu^+^ and Cu^0^ oxidation states makes the identification of Cu oxidation states difficult.^19^ Thus, we employed Auger electron spectroscopy (AES) to investigate the oxidation state of Cu. Both Cu_2_O and Cu_2_O@RF exhibited a single Cu LMM peak at ∼569.9 eV, indicating that the Cu_2_O and Cu_2_O@RF catalysts primarily consisted of the same Cu^+^ oxidation state. Therefore, the RF coating on Cu_2_O does not change the oxidation state of Cu. The charge density distribution of Cu_2_O@RF (Figure S4) also indicated that the RF coating does not alter the electronic state of Cu_2_O.

### Proton Transport Properties of RF Resins

**Figure 2:**
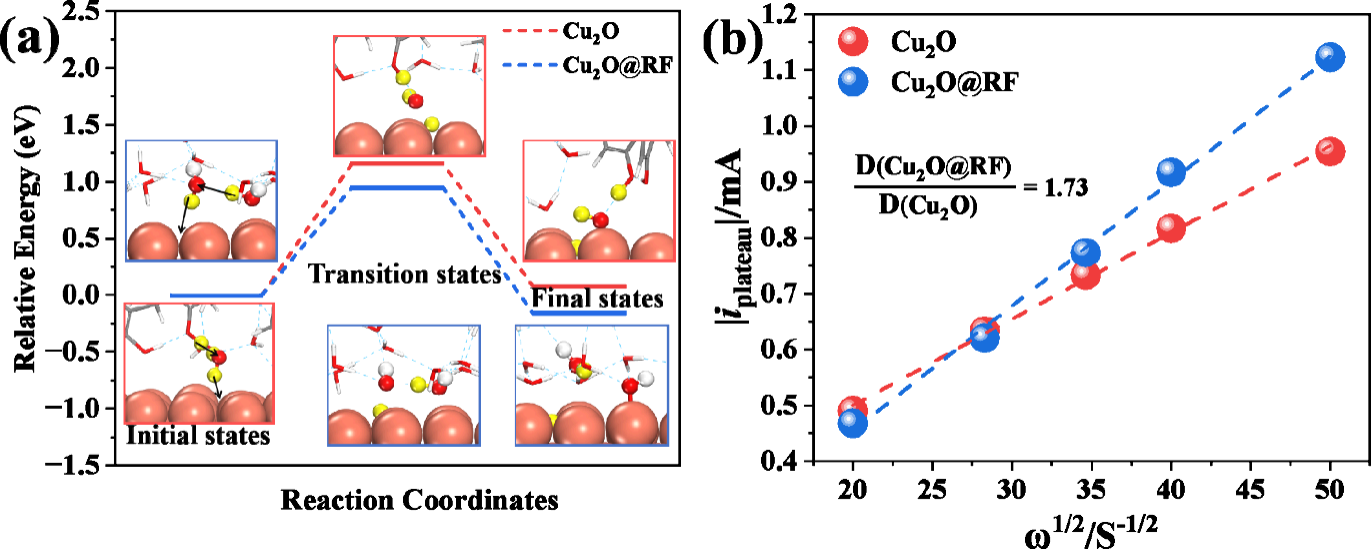
(a) Proton transfer energy barriers of Cu_2_O and Cu_2_O@RF. The inset pictures are calculated DFT models for Cu_2_O and Cu_2_O@RF. (The top three pictures are for Cu_2_O, and the bottom three pictures are for Cu_2_O@RF.) (b) Linear fitting of the plateau current vs ω^1/2^ on the Levich equation from LSVs of Cu_2_O@RF in N_2_-saturated 0.1 M KH_2_PO_4_ with different rotation rates.

To investigate the proton transport properties of RF resins, we simulated proton transport behaviors on a Cu surface in the presence and absence of an RF coating (Figure 2a). Without an RF coating, a proton is transported onto a Cu surface via an adjacent water molecule shuttle in a hydronium-like transition state. When RF is coated on the Cu surface, the RF layer becomes the proton shuttle locally since the proton can oscillate almost freely between the oxygen centers of the phenolic anions caused by hydrogen bonding.^20^ Protons are effectively delocalized over those oxygen centers, hence resulting in hyperacidity and an extremely fast proton transfer frequency^20^ (Scheme S3). Our DFT calculations (Figure 2a) also showed good agreement with those observations, indicating a lower energy barrier for proton transfer on a Cu surface covered with RF molecules than that of H_2_O molecules (0.95 vs 1.16 eV, respectively).

To determine the proton transfer rates of RF, we performed rotating disk electrode experiments on Cu_2_O and Cu_2_O@RF in N_2_-saturated 0.1 M KH_2_PO_4_. The results showed a plateau current from proton reduction for both samples (Figure S5).^21^ However, the plateau current for Cu_2_O@RF was found to be higher than that for Cu_2_O, which indicated that the RF coating enhanced proton reduction rates. Using the Levich eq (eqs S3 and S4), we estimated proton diffusion coefficients for both Cu_2_O@RF and Cu_2_O by a linear fitting of the plateau current vs rotation rate ω^1/2^ (Figure 2b). The proton diffusion coefficient of Cu_2_O@RF was found to be 1.73-fold higher than that of the Cu_2_O surface, suggesting that the RF is a more effective proton donor, which is consistent with DFT calculations.

### Effects of RF Resins on Electrocatalytic CO_2_RR

Electrochemical CO_2_RR measurements were conducted in CO_2_-saturated 0.1 M KHCO_3_ electrolytes using a gastight H-cell configuration. Over Cu_2_O nanocubes, ethylene and ethanol were the major products with negligible CH_4_ production (Figure S6). Those observations are consistent with previous studies; that is, Cu(100) facets favor the formation of C_2_ products.^22,23^ However, with the RF coating, Cu_2_O showed a significant shift in product selectivity, where the Faradaic efficiency of CH_4_ increased up to ∼60%. In contrast, the Faradaic efficiency of C_2_ products decreased from 61.5% to 18.8%. When RF was used as the catalyst alone, H_2_ was the sole product (Figure S7). The bulk electrolysis experiments have also been performed in Ar-saturated 0.1 M KHCO_3_ electrolyte. H_2_ was the main product, with a tiny amount of CO detected (Figure S8).

To exclude the possibility that CH_4_ generation stemmed from the reduction of residual formaldehyde used in the RF synthesis, we conducted the CO_2_RR over Cu_2_O@RF in the presence of formaldehyde (200 mM) in the electrolyte. A small amount of CH_4_ was observed while methanol became the major product (Figure S9). This is in line with previous studies of formaldehyde electroreduction, where methanol is the core product.^24^ Those results indicated that the formation of methane over Cu_2_O@RF catalyst is not due to the presence of residual formaldehyde, if any, but to the reduction of CO_2_.

The enhanced stability has been observed upon RF coating over Cu_2_O for CO_2_RR. As shown in Figure S10, Cu_2_O@RF exhibited that the current density remained stable, with the Faradaic efficiency of CH_4_ nearly unchanged in a 3 h measurement. However, the current density over Cu_2_O decreased by approximately 12% with a significant decrease in the Faradaic efficiency. Postcharacterizations after CO_2_RR showed that the Cu_2_O nanocubes had disintegrated into smaller particle aggregates, while for the RF-coated Cu_2_O sample, both the cubic morphological structure of Cu_2_O particles and the RF coating layer remained intact (Figure S11). Therefore, the RF coating can stabilize and protect Cu catalysts from (electro)chemical degradation during the electrochemical CO_2_ reduction process.^25^

**Figure 3:**
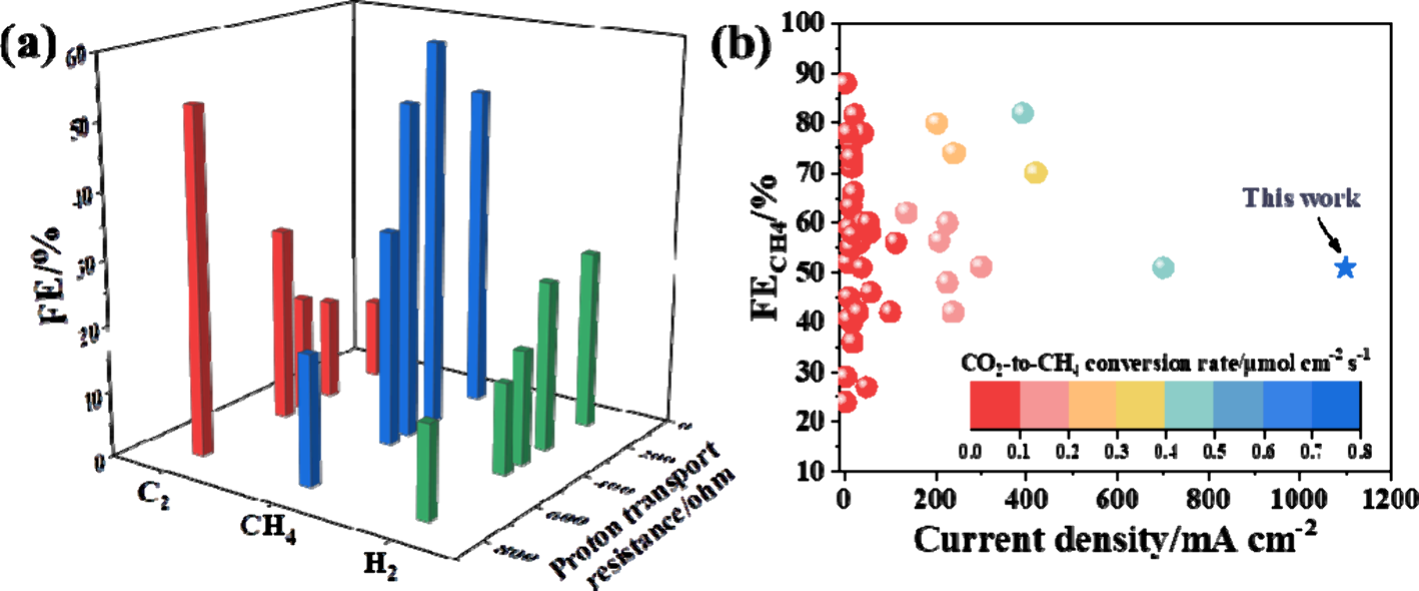
(a) Faradaic efficiencies of C_2_ compounds, CH_4_, and H_2_ versus the rate of proton transfer to Cu_2_O. (b) Comparison of our work with previous studies of the electrocatalytic CO_2_-to-CH_4_ reaction.

To investigate the effects of RF polymerization on proton transport and CO_2_RR, Cu_2_O@RF samples with an increasing degree of RF polymerization were prepared by increasing formaldehyde concentrations during catalyst synthesis. The polymerization degree of RF resins has been reported to increases with the concentration of formaldehyde as the rate of methylene linkages between resorcinol structures increases with the formaldehyde concentration,^26,27^ leading to the formation of higher-molecular-weight RF resins. The proton transfer rates were investigated by using electrochemical impedance spectroscopy (EIS) under humid conditions; an illustration of the EIS experiments is presented in Figure S12. The EIS results showed that the proton transfer resistances over Cu_2_O@RF catalysts decreased with an increasing degree of RF polymerization (Figure S13). The Cu_2_O@RF catalysts were also evaluated for their corresponding CO_2_RR performances. The Faradaic efficiencies of various CO_2_RR products at different potentials are shown in Figure S14, and Figure 3a shows the relationships between the Faradaic efficiencies of CO_2_RR products and the proton transfer resistances over the Cu_2_O@RF catalysts. As the degree of RF polymerization increased, resulting in a decrease in local proton transfer resistances (that is, the increase in local proton transfer rates), product selectivities shifted significantly from C_2_ products to CH_4_. The selectivity of CH_4_ increased from 19% to 58% while C_2_ product selectivity decreased from 52% to 15%. However, with further increases in RF polymerization and thus proton transfer rates, the Faradaic efficiency of CH_4_ decreased to 49% while the Faradaic efficiency of H_2_ increased to 27%.

Hence, optimizing RF polymerization is effective for modulating local proton transfer rates near the catalyst surface and achieving a high CH_4_ selectivity. During CO_2_RR, the local pH increases near the electrode surface due to the consumption of protons, which is linearly correlated with current densities.^28^ A high local pH environment has been shown to suppress the formation of CH_4_ and H_2_ and correspondingly increases C_2_H_4_ selectivity.^28^ We found, however, with an optimized RF coating layer on the Cu surface that protons can be efficiently replenished locally at the electrode surface, resulting in high CH_4_ selectivity.

When Cu_2_O@RF was utilized in flow electrolyzers with gas diffusion electrodes to overcome the large intrinsic mass transfer limitations of CO_2_ in H-cells, the Faradaic efficiency of CH_4_ could reach as high as ∼51% at a high current density of 1.1 A cm^–2^ (Figures 3b and S15). The corresponding CO_2_-to-CH_4_ conversion rate was 0.72 μmol cm^–2^ s^–1^, which is higher than in any previous study to the best of our knowledge (Figure 3b and Table S1). In contrast, Cu_2_O showed a C_2_ product selectivity of ∼53% at a current density of 300 mA cm^–2^ and ∼72% at 700 mA cm^–2^ (Figure S15).

### Mechanism Investigations

**Figure 4:**
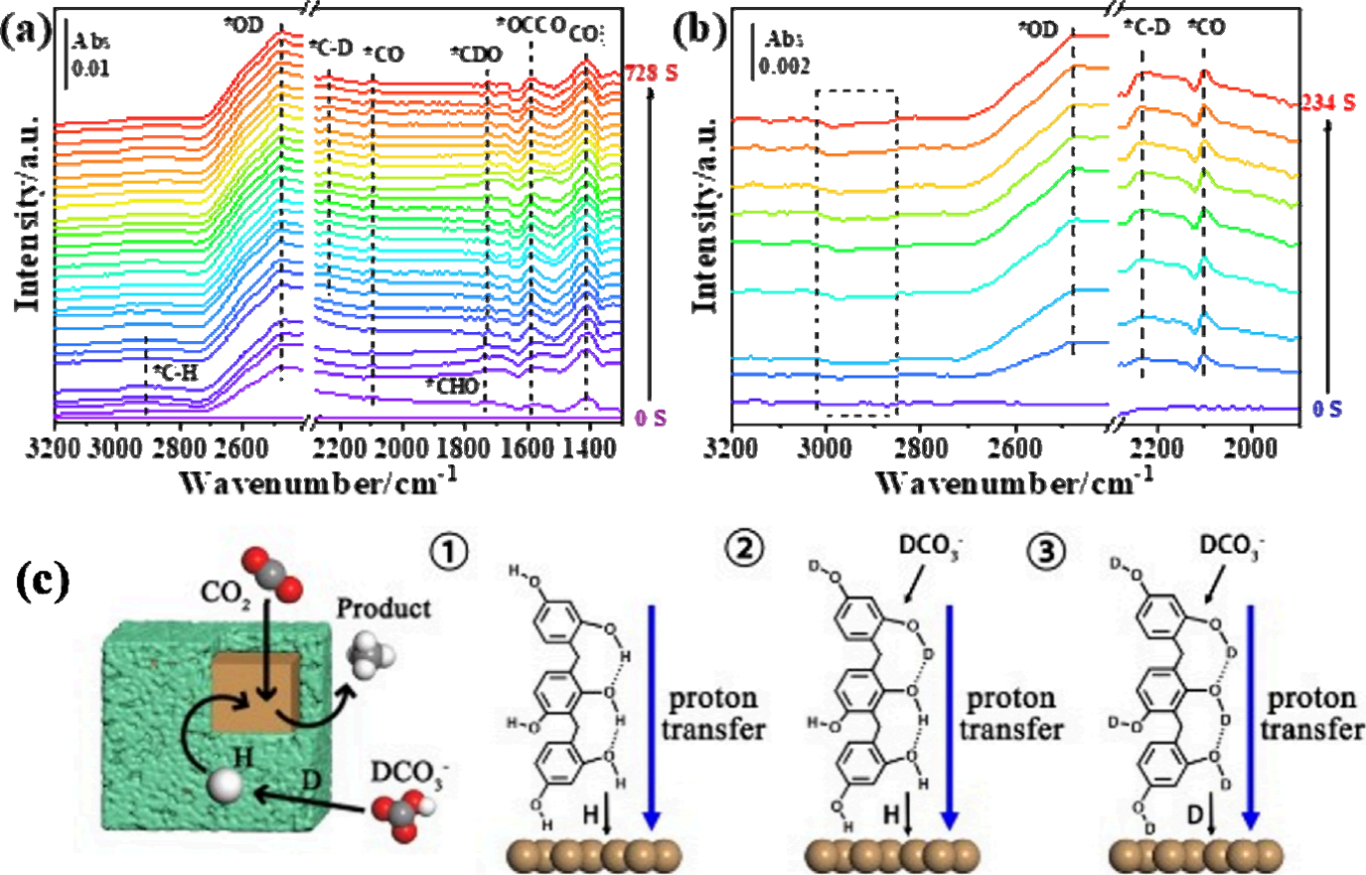
*In situ* ATR-FTIR spectra of (a) Cu_2_O@RF and (b) Cu_2_O along the reaction time at −1.2 V vs RHE in CO_2_-saturated 0.05 M K_2_CO_3_/D_2_O solutions. (c) Schematic of proton transfer in Cu_2_O@RF.

*In situ* ATR-FTIR spectroscopy was conducted to investigate the CO_2_RR mechanisms and pathways over the Cu_2_O@RF and Cu_2_O catalysts. Figure S16 shows the ATR-FTIR spectra of Cu_2_O@RF and Cu_2_O at various potentials in CO_2_-saturated 0.1 M KHCO_3_/H_2_O electrolytes. The *OCCO intermediate at 1540 cm^–1^ over Cu_2_O indicated that Cu_2_O exhibited active sites for C–C coupling for the formation of C_2_ products, while Cu_2_O@RF did not show the *OCCO peak. Figure 4 shows the ATR-FTIR spectra of Cu_2_O@RF and Cu_2_O at various potentials in CO_2_-saturated 0.1 M KHCO_3_/D_2_O electrolytes. Two C–H vibrational bands are clearly visible for Cu_2_O@RF due to the adsorption of *CH_*x*_ on Cu_2_O@RF for the formation of CH_4_, while the *C–H bands are absent over Cu_2_O since it favors C_2_H_4_ generation rather than CH_4_.

To further investigate the proton transfer process, 0.05 M K_2_CO_3_/D_2_O solutions were utilized as the electrolytes, which change to 0.1 M KDCO_3_ in D_2_O after CO_2_ saturation. Figure 4 shows the ATR-FTIR spectra over Cu_2_O@RF and Cu_2_O collected at −1.2 V vs RHE at various time intervals. The *C–H and *CHO vibration bands at around 2900 and 1730 cm^–1^, respectively, were initially observed on the Cu_2_O@RF catalyst (Figure S17a). However, as the reaction time increased, the *C–H band and *CHO band gradually vanished, while the *C–D band and *CDO band emerged. For Cu_2_O, throughout the reaction times, only the *C–D band was observed (Figure S17b).

Figure 4c shows a proposed proton transfer process of Cu_2_O@RF derived from the ATR-FTIR spectroscopic results. Initially, protons in *CH and *CHO originated from the RF. Once those protons were consumed, deuterium participated in the reaction through the proton shuttling mechanism of the RF. The p*K*_a_ of phenol is 9.95. The acidity of the OH groups of the phenolic resins is considerably higher than that of phenol and increases with the polymerization degree.^20^ The higher acidity of RF compared to that of H_2_O and HCO_3_^–^ caused RF to deprotonate more readily than H_2_O or HCO_3_^–^.^29^ Moreover, RF resins possess significant proton capacity due to the presence of a large number of hydroxyl groups in the phenolic resins. As a result, hydrogen/deuterium is favored to transfer through the RF layers driven by the proton concentration gradient between the catalyst/electrolyte interface and the bulk electrolyte, hence enabling the RF to shuttle protons effectively from the electrolyte to the electrode surface in the CO_2_RR.

Moreover the kinetic isotope effect was measured to evaluate the reaction kinetics of the protonation process. If the proton transfer is involved in the rate-determining step (RDS), a KIE value of 2–7 is expected, named as the primary KIE.^30−32^ In the case of Cu_2_O, the KIE values for H_2_, CO, CH_4_, and C_2_H_4_ were 4.8, 0.8, 5.5, and 1.0, respectively (Figure S18). The results indicate that the RDS of CH_4_ and H_2_ involves proton transfer but C_2_H_4_ does not. Upon RF coating, the KIE value of methane formation decreased to 1.8, while that of hydrogen generation dropped to 3.2. The decrease in KIE values over Cu_2_O@RF could be attributed to the enhanced proton transfer. In addition, the KIE is usually utilized as a feature to determine whether a reaction follows a proton-coupled electron transfer (PCET) process or a sequential proton–electron transfer (SPET) process.^33,34^ Overall, the KIE value for C_2_H_4_, which is close to 1, suggests that the rate-determining step of C_2_H_4_ is not a PCET process, while CH_4_ formation proceeds through a PCET process.

To investigate the effect of proton transfer on products selectivity, we calculated the reaction pathways for CH_4_ and C_2_ product formation over Cu(100) and Cu(100)@RF surfaces (Figures S19–S22) (for Cu_2_O and Cu_2_O@RF, respectively). The CO_2_ is converted to the CO intermediate over Cu before undergoing further reduction. Thus, we focus on CO rather than CO_2_ as a starting point. For CH_4_ formation on Cu_2_O and Cu_2_O@RF, the rate-determining step is the same CO* + H*→ CHO* step with the activation barriers of 1.34 and 0.97 eV, respectively, which involves proton transfer via a PCET path. The CO* chemisorbs at the Cu surfaces, and simultaneously, a proton transferred from the approaching H_2_O molecules or RF molecules reacts with CO* to form CHO*, concomitant with an electron transfer from the surface. Similarly, the subsequent formation of CHOH* results from the reaction of CHO* and a proton transferred from the H_2_O/RF molecules in a PCET process. The activation barrier of CHOH* formation on Cu_2_O is 1.07 eV, which is 0.16 eV higher than that on Cu_2_O@RF for this PCET process. The results indicate that promoting the proton transport rate could efficiently reduce the barriers of the PCET process. As shown in Figure S19, the CO* coupling is the potential-determining step on Cu_2_O and Cu_2_O@RF surfaces for C_2_ products. Since Cu_2_O has a lower free energy for CO* coupling than Cu_2_O@RF, Cu_2_O showed a higher activity for C_2_ products.

**Figure 5:**
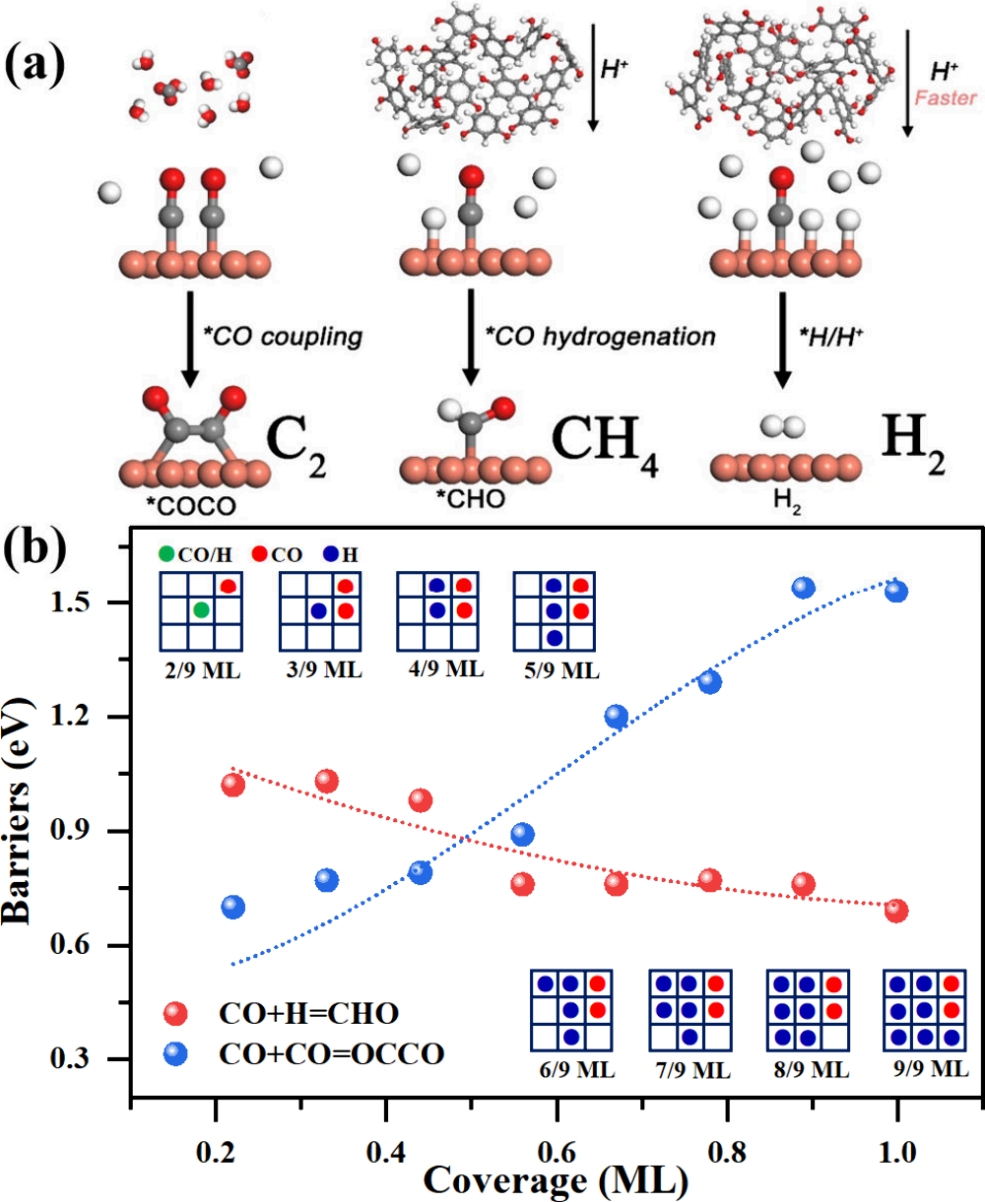
(a) Diagram of the relationship between the proton transfer rate and product selectivity (white, gray, and red balls represent hydrogen, carbon, and oxygen atoms, respectively). (b) Energy barriers of CO hydrogenation and coupling processes with different hydrogen coverage situations.

The relationship between proton transfer rates and product selectivities is summarized in Figure 5a. The Tafel relation of ethylene generation is pH-independent and the RDS is *CO/*CHO dimerization,^35,36^ which does not involve proton transfer. Under proton-deficient conditions, ethylene is the major product. As the proton transfer rate increases, more protons arrive at the reaction surface; thus, proton-coupled electron transfer dominates and CO hydrogenation occurs to generate CH_4_. If the proton transfer rate further increases, the HER reaction will dominate.

Taking the solvent model as an example, we further investigated the effect of the proton transfer rate on product selectivity and assumed that an increase in the proton transfer rate would result in an increase in the coverage of H atoms on the catalyst surface. Assuming that there are nine adsorption sites on a 4 × 4 Cu(100)-48H_2_O surface slab and based on the most stable adsorption configurations of CO and H species (both on the four Cu acupoints), the increase in H atom coverages would lead to an enhanced interaction between CO and H species, which would significantly increase the barriers for CO coupling, as shown in Figures 5b and S22. However, the formation of CHO* is less vulnerable to adsorbate interaction effects due to its smaller spatial structure; hence, the activation energy of CO hydrogenation has no noticeable change with increasing coverage. The above results are consistent with the observation that RF is effective for enhancing proton transfer to induce CH_4_ production.

## Conclusions

This study presents a facile strategy to regulate proton transport rates utilizing the surface resin layer, efficiently tuning product selectivities between methane and ethylene over cuprous oxides during the CO_2_RR. This proton transfer manipulation enables appreciable hydrogen coverage for methane generation, which achieves a remarkably high current density of 1 A cm^–2^ with over 50% faradaic efficiency. The chemical microenvironment regulation provides a facile method to control electrochemical reactions, which could be further extended to other electrochemical reactions, especially for those involving protons.

## Supporting Information Available

Experimental details, Levich equation, TEM images, XRD patterns, in situ ATR-FTIR spectra, electrochemical performance, and DFT calculations (PDF)
