# Supplementary material for: Proton-Transfer Dynamics Regulates CO2 Electroreduction Products via Hydrogen Coverage
Source: ACS Cent Sci. 2024 Nov 28;10(12):2331–7. doi: 10.1021/acscentsci.4c01534 (PMC11672531; doi:10.1021/acscentsci.4c01534)
Supplement: Supplementary file 1 — oc4c01534_si_001.pdf [file oc4c01534_si_001.pdf]

Supplemental information

Proton transfer dynamics regulates CO_2 electroreduction products via hydrogen coverage

Qun Fan[1†], Tiantian Xiao [1†], Hai Liu[1], Tianxiang Yan[1], Jianlong Lin[1], Siyu Kuang[1], Haoyuan Chi[1], Thomas J. Meyer[3], Sheng Zhang[1,2,], Xinbin Ma[1,2]*

1 Key Laboratory for Green Chemical Technology of Ministry of Education, Collaborative Innovation Centre of Chemical Science and Engineering, School of Chemical Engineering and Technology, Tianjin University, Tianjin 300072, China.

2 Haihe Laboratory of Sustainable Chemical Transformations, Tianjin 300192, China.

3 Department of Chemistry, University of North Carolina at Chapel Hill, Chapel Hill, North Carolina 27599, United States.

† These authors contributed equally to this work.

E-mail: sheng.zhang@tju.edu.cn

Experimental Section

Materials Synthesis

Cu_2O nanocubes In a typical procedure, 1 mmol $CuCl_2 \cdot H_2O$ was dissolved in 400 mL distilled water at 25 °C, then the NaOH solution (40.0 mL, 0.10 M) were added under constant magnetic stirring, and blue $Cu(OH)_2$ precipitates appeared immediately. After the solution were stirred for about 5 min, ascorbic acid solution (40.0 mL, 25 mM) was added and keep 30 min. Then the precipitates were separated by suction filtration and washed with distilled water and absolute ethanol for several times.

Cu_2O@RF 100 mg Cu_2O were fist dispersed in 10 mL ethanol, which were subjected to bath ultrasonication for 20 min. Then 25 mL NaOH solution (0.05 mg mL^{-1}) were added into the above dispersion at 70 °C under magnetic stirring. Formaldehyde (37%, 737 μL) and resorcinol (548 mg) were added into the mixture and stirred for 24 hours. The precipitates were centrifuged and washed with distilled water and absolute ethanol for several times, then dried at 80 °C. The amounts of formaldehyde changed from 246 μL, 369 μL, 491 μL, 737 μL, to 983 μL.

Cu The synthesis process was similar to Cu_2O, except that the concentration of ascorbic acid was changed to 5 M.

Cu@RF The synthesis process was similar to Cu_2O@RF, except using Cu to replace Cu_2O.

CuO 150 mg Cu_2O were placed in a muffle furnace with 10 g DI water, heated to 300 ℃ at a rate of 5 ℃/min in an air.

CuO@RF The synthesis process was similar to Cu_2O@RF, except using CuO to replace Cu_2O.

Cu_2O@BF 100 mg Cu_2O were fist dispersed in 10 mL ethanol, which were subjected to bath ultrasonication for 20 min. Then 687.6 mg 3-hydroxybenzoic acid was added in the above dispersion. NaOH solution (0.05 mg mL^{-1}) was used to tune the pH of the

above solution until 9. Formaldehyde (37%, 737 μL) were added into the mixture and stirred at 70 °C for 24 hours. The precipitates were centrifuged and washed with distilled water and absolute ethanol for several times each, then dried at 80 °C.

Materials Characterization

X-ray powder diffraction (XRD) was performed with a D8 Advance diffractometer (Bruker) equipped with a Cu Kα radiation. X-ray photoelectron spectroscopy (XPS) experiment was carried out using photoelectron spectrometer (K-Alpha+) with a monochromatic Al Ka X-ray (1486.6 eV) source. All spectra were calibrated according to the C 1s binding energy at 284.8 eV. Transmission electron microscope (TEM) was conducted using JEOL-1400F microscope and JEOL ARM200F aberration-corrected high-angle annular dark field scanning transmission electron microscope. TEM samples were prepared by depositing a droplet of suspension onto a Cu grid coated with a Lacey Carbon film. Low-pressure N_2 adsorption–desorption isotherms for the Brunauer–Emmett–Teller (BET) specific surface area were recorded on an Autosorb-iQ (Quantachrome) analyzer at 77 K.

Measurements for CO_2RR

Electrode Preparation Typically, a catalyst (10 mg) was dispersed in 5 mL isopropanol and 10 μL Nafion solution (5 wt%) under bath ultrasonication for 30 min to form a homogeneous ink. Some amounts of the dispersion (500 μL) were then loaded onto a carbon paper electrode with an area of 1 cm x 1 cm, which was then dried under heating lamp.

Electrochemical measurements Controlled potential electrolysis of CO_2 was tested in a custom-made gas-tight H-cell system, which was separated by a Nafion 117 membrane. Toray Carbon fiber paper with a size of 1 cm × 1 cm was used as working electrode. Pt wire and Ag/AgCl electrodes (filled with saturated KCl aqueous solution) were used as counter electrode and reference electrode, respectively. The potentials

were controlled by an electrochemical working station (CHI 760E, Shanghai CH Instruments Co., China). All potentials in this study were measured against the Ag/AgCl reference electrode and converted to the RHE reference scale by

E (vs. RHE) = E (vs. Ag/AgCl) + 0.21 V + 0.0591 × pH (Eq. S1)

Electrocatalytic CO_2 reduction was conducted in CO_2-saturated $KHCO_3$ solution (0.1 M) at room temperature and atmospheric pressure. CO_2 was purged into the $KHCO_3$ solution for at least 30 min to remove residual air in the reservoir.

Faradaic efficiency (FE) measurements Based on the definition of faradaic efficiency

$$FE = Q_{CO}/Q_{total} = (Z \times n \times F)/Q_{total} \quad \text{(Eq. S2)}$$

where Z is the number of electrons transferred, n the number of moles for a given product, F Faraday's constant (96 485 C mol^{-1}), Q_{total} all the charge passed throughout the electrolysis process (measured by calculating the curve area of current density *vs.* time plot).

Flow-cell Test Flow cell tests were performed in a custom-made reactor made of poly ether ether ketone (PEEK). YLS-30T Carbon paper coated catalysts (1 mg cm^{-2}) was used as the working electrode. Ag/AgCl and a Pt foil was used as the reference and anode electrode, respectively. A piece of cation exchange membrane (Fumatech) was used as the separator. The electrolyte was 1 M $KHCO_3$. During the tests, CO_2 was supplied from the back of GDL with a flow rate of 30 sccm controlled by a gas mass flowmeter (Horiba). The catholyte was forced through peristaltic pump to continuously circulate with a flow rate of 30 ml min^{-1}. The anolyte was circulated by a gas-liquid mixing pump to reduce the disturbance of the produced gas.

In situ attenuated total reflection Fourier transform infrared spectroscopy (FTIR) characterizations The operando FTIR measurements were performed with Thermo Fisher Nicolet iS50. The measurements were conducted in a home-made 3-electrode spectroelectro-chemical cell. Ag/AgCl electrode and Pt wire were used as the reference and counter electrode, respectively. Silica pillared sprayed gold for 50 S, then catalyst

ink dropped on silica pillared as working electrode. The accumulation time for each spectrum were 30 s. During each experiment, CO_2 was bubbled through the electrolyte.

Rotating Disk Electrode (RDE) Experiments RDE experiments were performed using a Pine Research MSR Rotator in a 0.1 M KH_2PO_4 electrolyte under N_2. Glass carbon electrodes were mechanically polished with 3 μm, and 0.1 μm Al_2O_3 powders, and finally sonicated in DI water for 5 minutes before use. A carbon rod was used as the counter electrode and an Ag/AgCl was used as the reference electrode. Cyclic voltammograms were collected at 400, 800, 1200, 1600, and 2500 rpm.

$$I_L = (0.062)nFAD^{\frac{2}{3}}\omega^{\frac{1}{2}}v^{-\frac{1}{6}}C \quad \text{(Eq. S3)}$$

where I_L is the Levich current (A), n is the number of moles of electrons transferred in the half reaction (number), F is the Faraday constant (C/mol), A is the electrode area (cm^2), D is the diffusion coefficient (see Fick's law of diffusion) (cm^2/s), ω is the angular rotation rate of the electrode (rad/s), v is the kinematic viscosity (cm^2/s), C is the analyte concentration (mol/cm^3).

$$\frac{D_{Cu_2O@RF}}{D_{Cu_2O}} = \left(\frac{slope_{Cu_2O@RF}}{slope_{Cu_2O}}\right)^{\frac{3}{2}} = 1.73 \quad \text{(Eq. S4)}$$

where $D_{Cu2O@RF}$ is the diffusion coefficient of Cu_2O@RF, D_{Cu2O} is the diffusion coefficient of Cu_2O, $slope_{Cu2O@RF}$ is the slope of Cu_2O@RF, $slope_{Cu2O}$ is the slope of Cu_2O. Note that the D discussed here reflects overall effects of the film on the proton mass transfer at the electrode interface, rather than the absolute H^+ diffusion coefficient in the solution.[1]

DFT calculation All calculations were calculated with density functional theory using the Perdew-Burke-Ernzerhof (PBE) generalized gradient approximation (GGA) functional in Vienna Ab initio Simulation Package (VASP).[2] All geometries were fully optimized with a plane wave energy cutoff of 400 eV and the electronic energy and forces were converged to within 10^{-5} eV and -0.03 eV/Å, respectively. The climbing image nudged elastic band (CI-NEB) was used to search transition state, and the results are further confirmed using frequency analysis.[3] For all active energies, the zero point energy (ZPE) correction was carried out[4]: $ZPE = \sum_i \frac{1}{2} h v_i$,Where h and v_i denote the

Plank constant and the computed real frequencies. The activation and reaction Gibbs free energies were calculated by including zero-points energies, thermal corrections and entropy terms at 298 K. The CHE model (at 0 V vs RHE) proposed by Nørskov et al. was determined the potential-dependent reaction and activation free energies.[5]

We constructed a complex liquid/electrode interface is essential to include to the effect of solvents, a five explicit layers of water molecules(48 molecules, 1.21 nm thick) on 4*4 Cu(100) surface slab(three layers) with an area of 1.02 nm^2 is described the water/Cu(100) interface.[6] For Cu(100)-RF, we determined that one resorcinol would be present between the Cu(100) surface and water layers. The 10 ps of AIMD simulations were carried out at 298 K for two model. It should be point that the RF molecule remain mobile near the Cu(100) surface, thus the total energy of system is fluctuating. The Brillouin zone was sampled by 1*1*1 Monkhorst-Pack k-points sampling for above models.

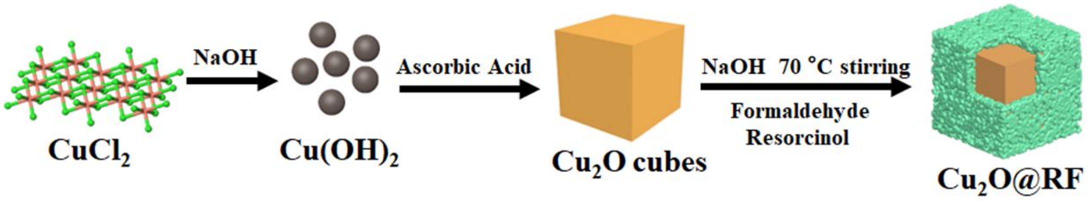

Scheme S1. Illustration of synthetic route of Cu_2O@RF.

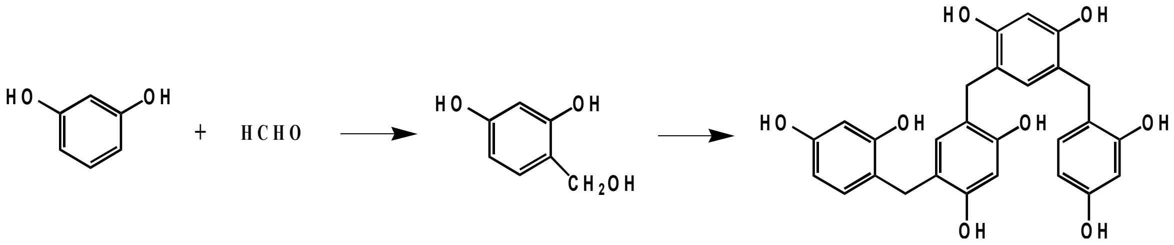

Scheme S2. Illustration of resin polymerization from formaldehyde and resorcinol.

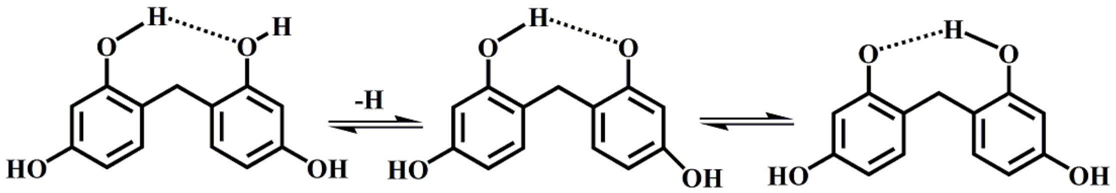

Scheme S3. Illustration of proton transfer in RF.

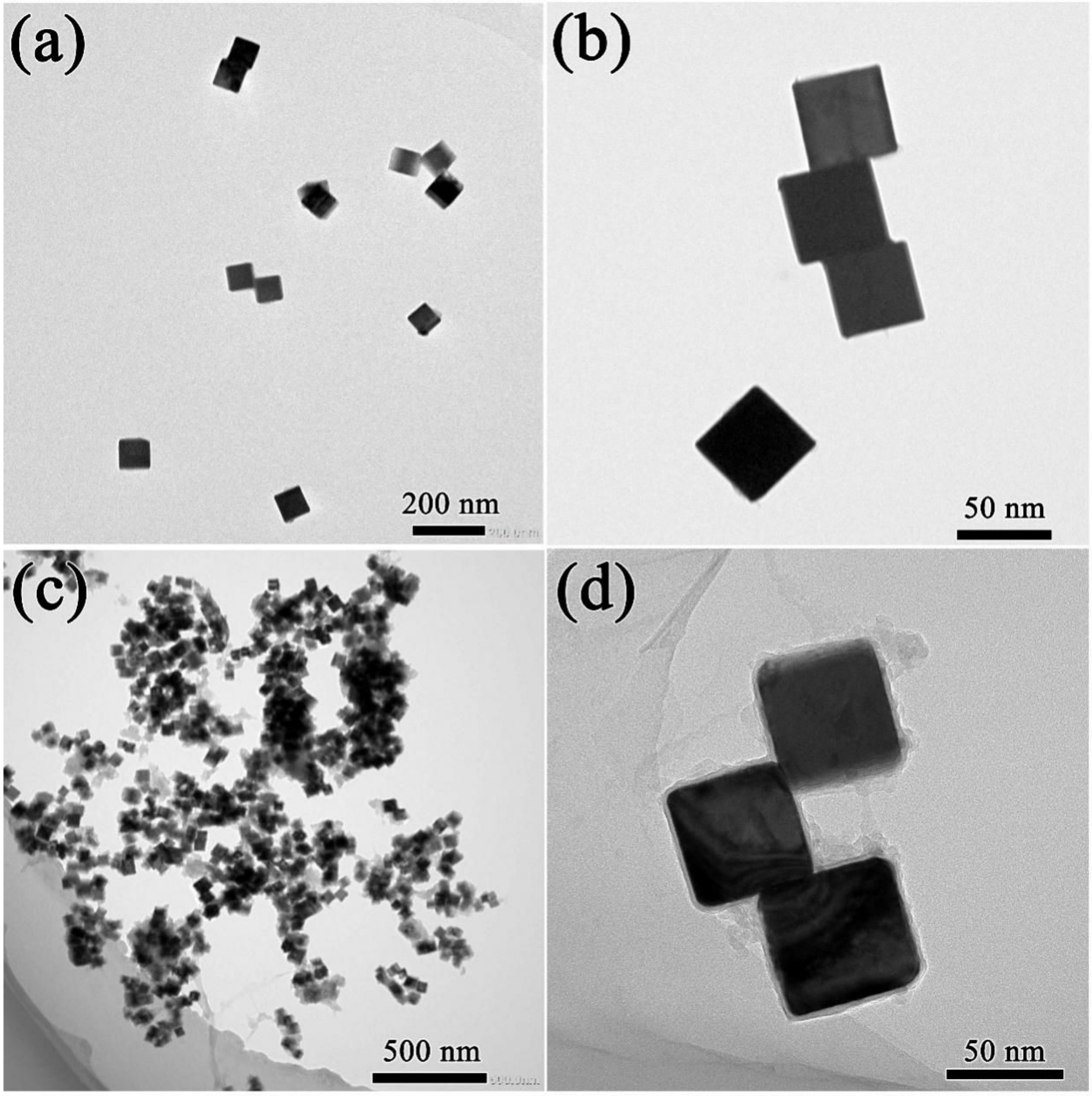

Figure S1. TEM images of (a-b) Cu_2O and (c-d) Cu_2O@RF.

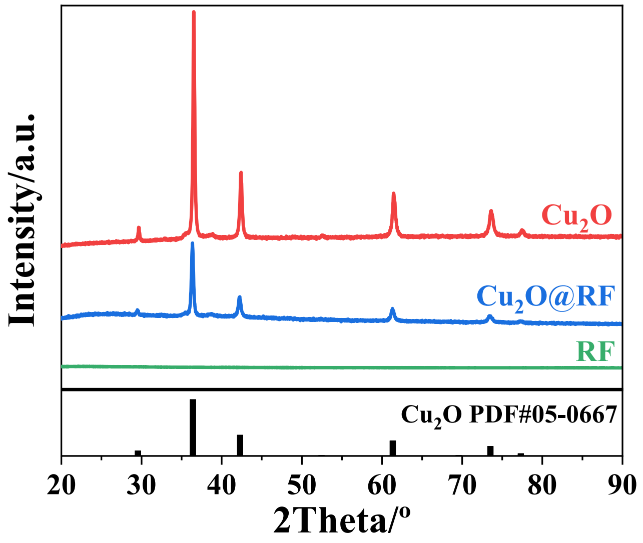

Figure S2. XRD pattern of Cu_2O, Cu_2O@RF and RF.

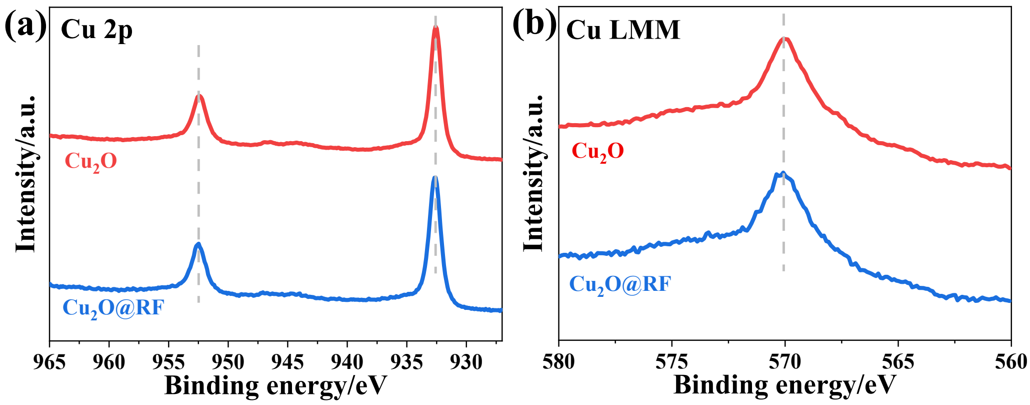

Figure S3. (a) XPS Cu 2p spectra and (b) Cu LMM auger spectra of Cu_2O and Cu_2O@RF.

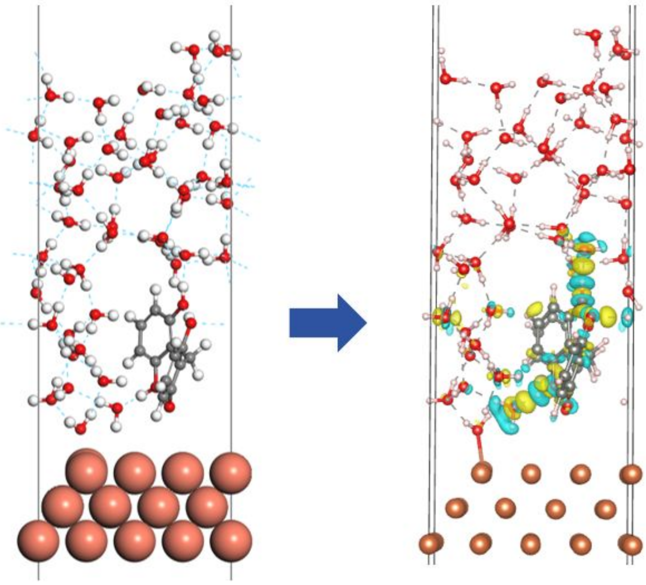

Figure S4. The charge density difference distribution for Cu_2O@RF. Note: isosurface=0.002.

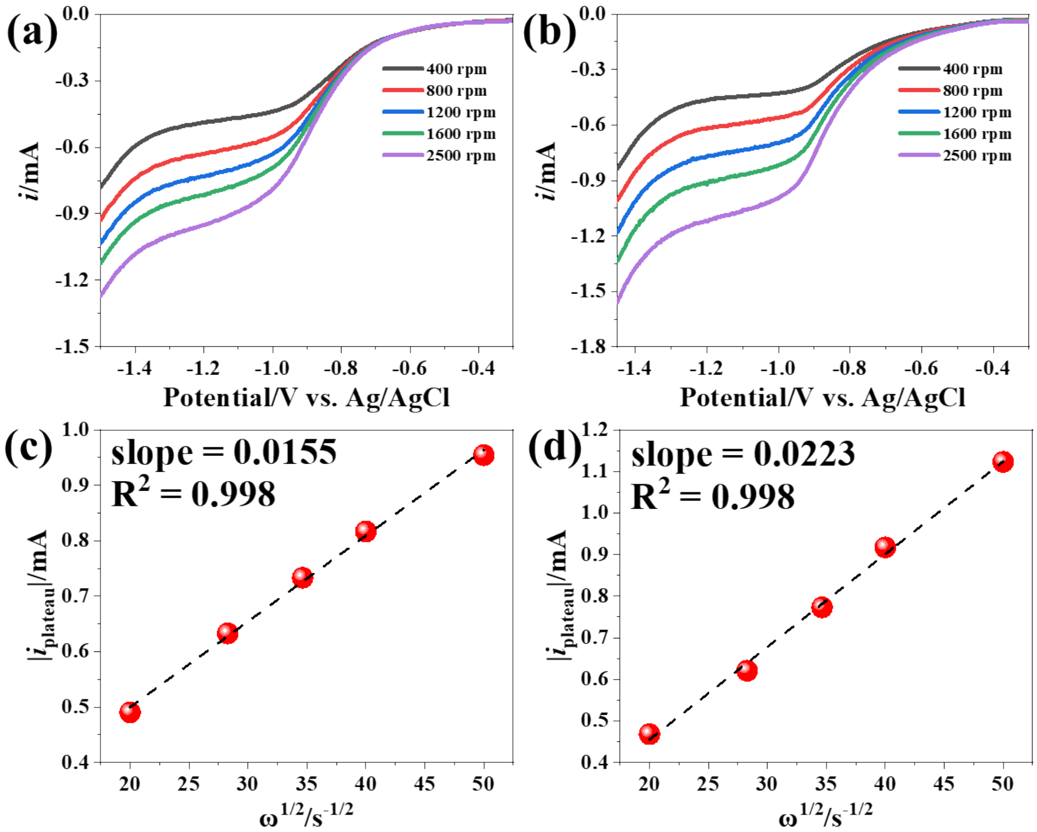

Figure S5. LSVs of (a) Cu_2O and (b) Cu_2O@RF in Ar-saturated 0.1 M KH_2PO_4 with different rotation rates, and linear fitting of plateau current vs. $\omega^{1/2}$ using Levich equation for (c) Cu_2O and (d) Cu_2O@RF.

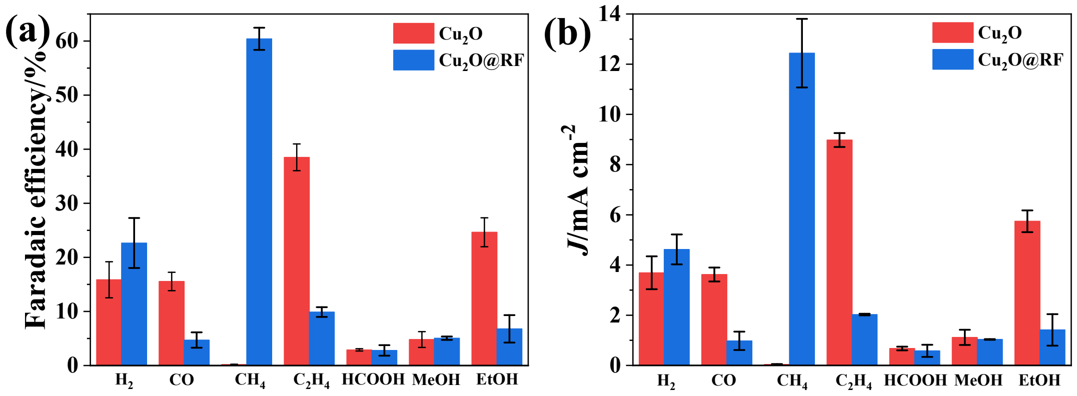

Figure S6. Comparison of (a) Faradaic efficiency and (b) partial current densities of various products at -1.8 V versus RHE between Cu_2O and Cu_2O@RF.

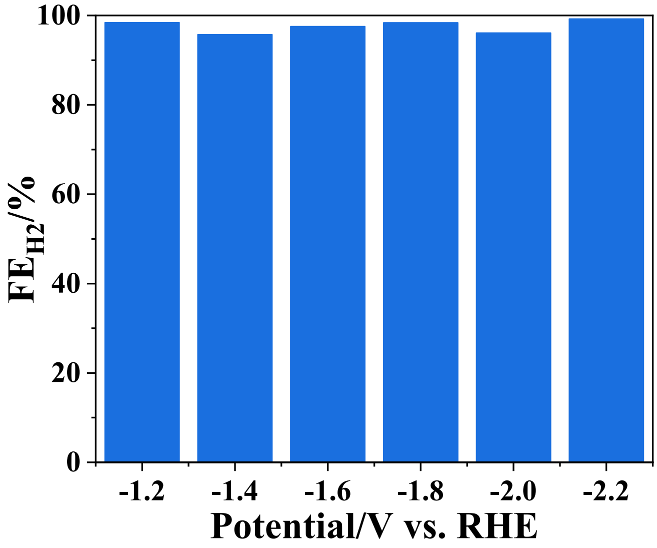

Figure S7. Faradaic efficiency of RF at various potentials.

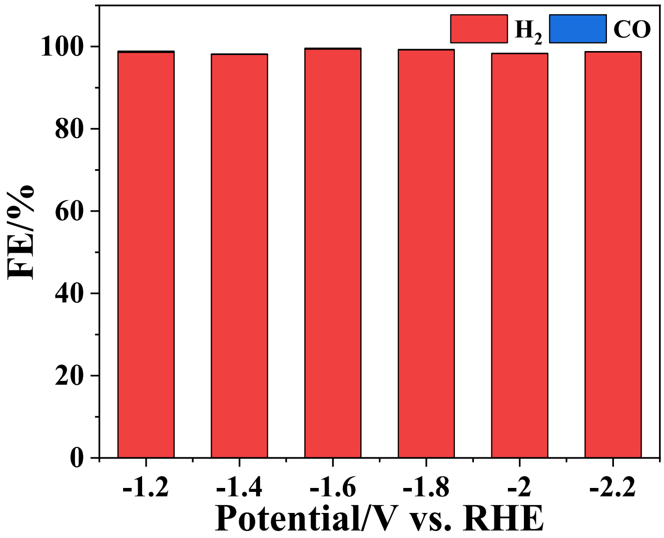

Figure S8. Faradaic efficiency of Cu_2O@RF in Ar environment at various potentials.

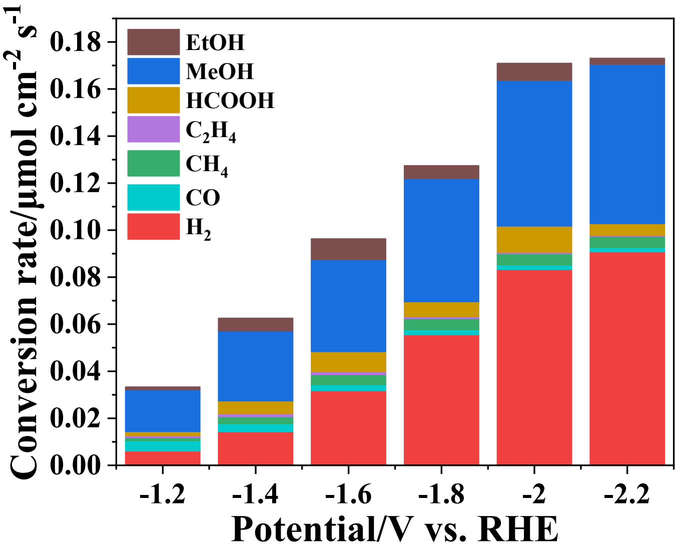

Figure S9. Conversion rate to various products at different potentials for Cu_2O@RF in CO_2-saturated 0.1 M $KHCO_3$ aq. electrolyte containing 200 mM HCHO.

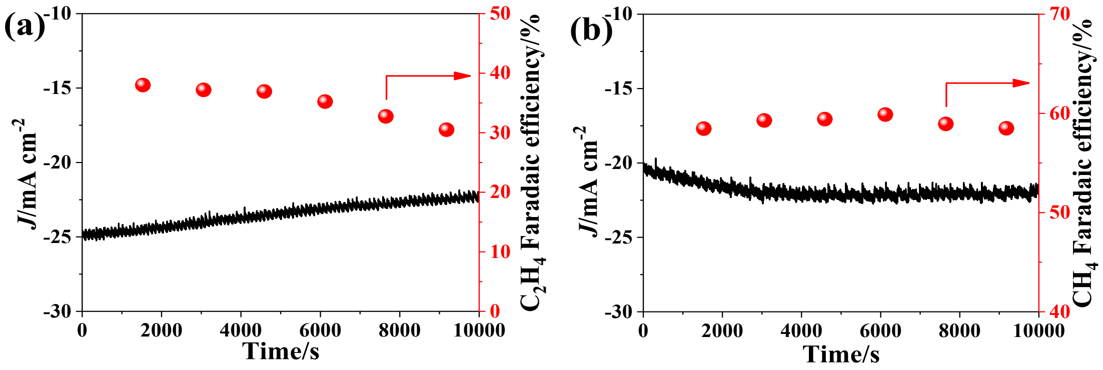

Figure S10. The change in the current density and the Faradaic efficiency of main product of the CO_2RR at -1.8 V vs. RHE for (a) Cu_2O and (b) Cu_2O@RF during a test period of 10000 s.

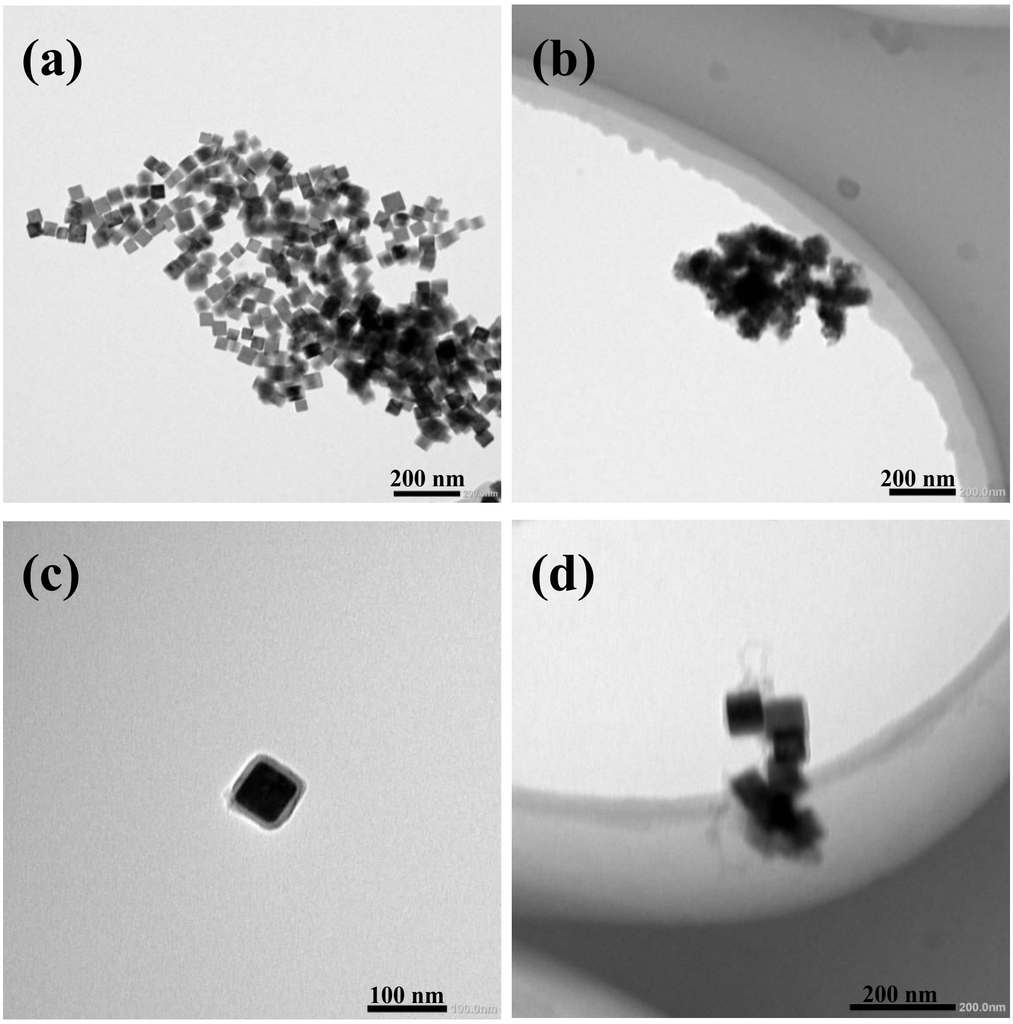

Figure S11. TEM images of Cu_2O before (a) CO_2RR, (b) after CO_2RR, (c) Cu_2O@RF before CO_2RR, and (d) Cu_2O@RF after CO_2RR.

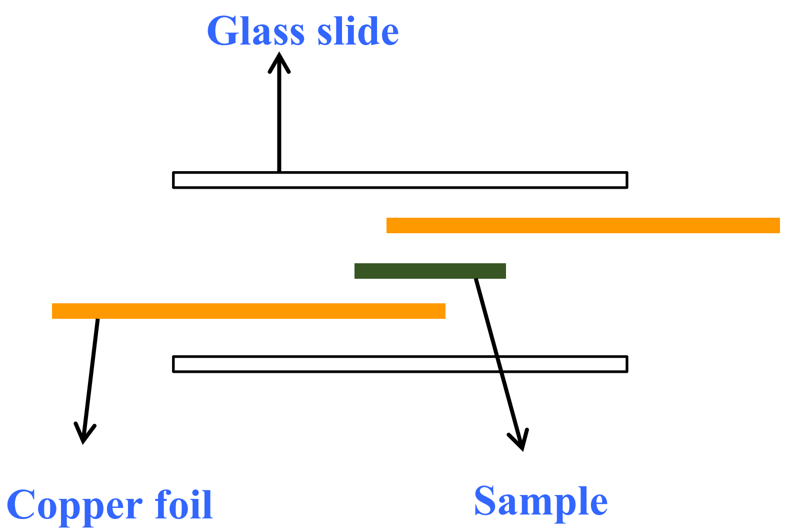

Figure S12. Illustration of electrochemical impedance spectroscopy device.

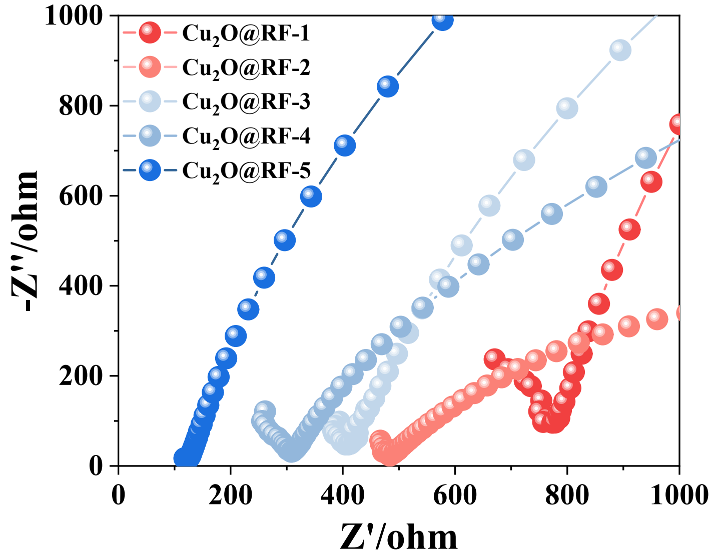

Figure S13. (a) The Nyquist plots and (b) comparison of various products Faradaic efficiencies at -1.8 V vs. RHE among different Cu_2O@RF samples.

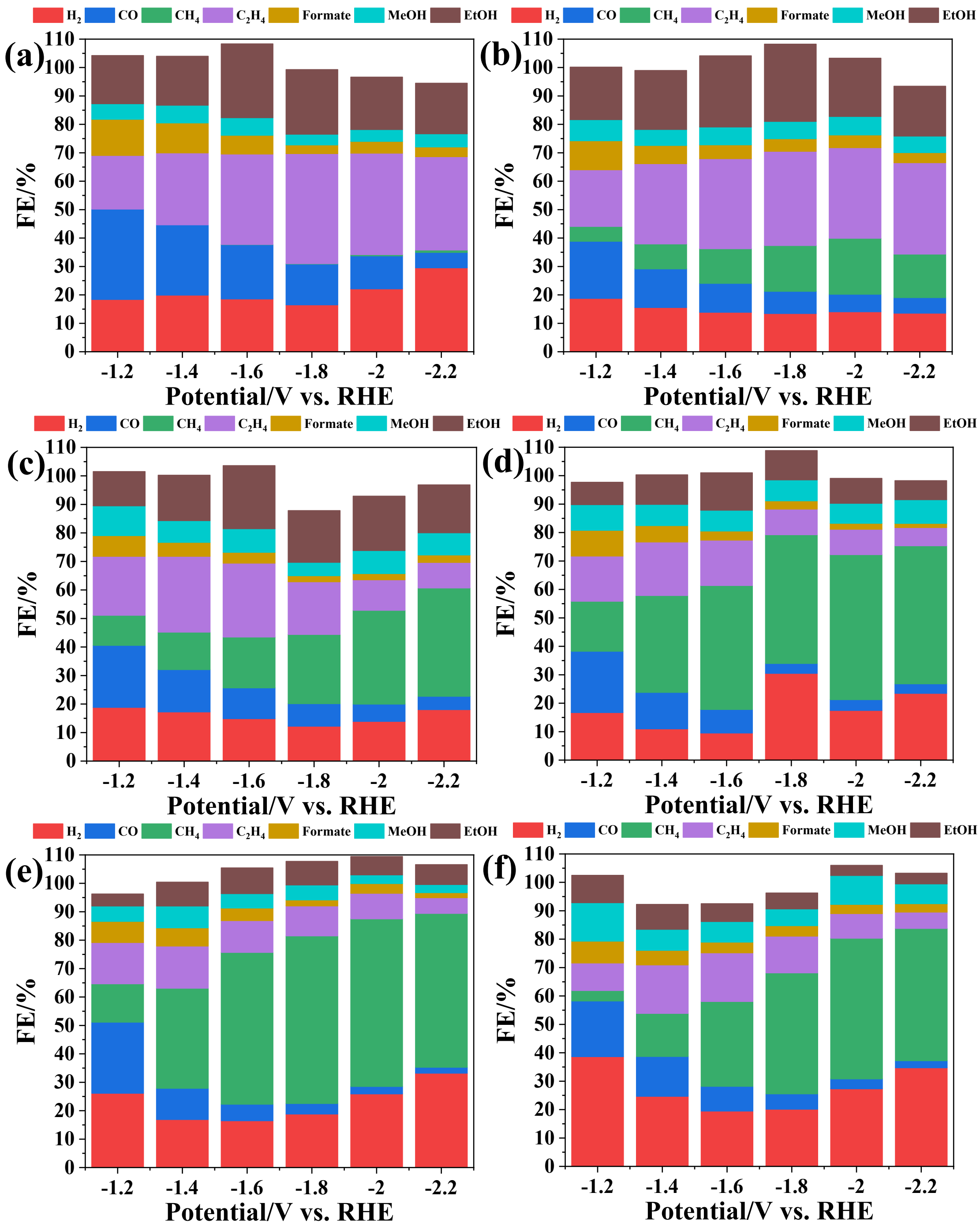

Figure S14. Faradaic efficiencies at various potentials over bared Cu_2O (a), Cu_2O@RF samples prepared with different amounts of formaldehyde 246 μL (b), 369 μL (c), 491 μL (d), 737 μL (e), and 983 μL (f).

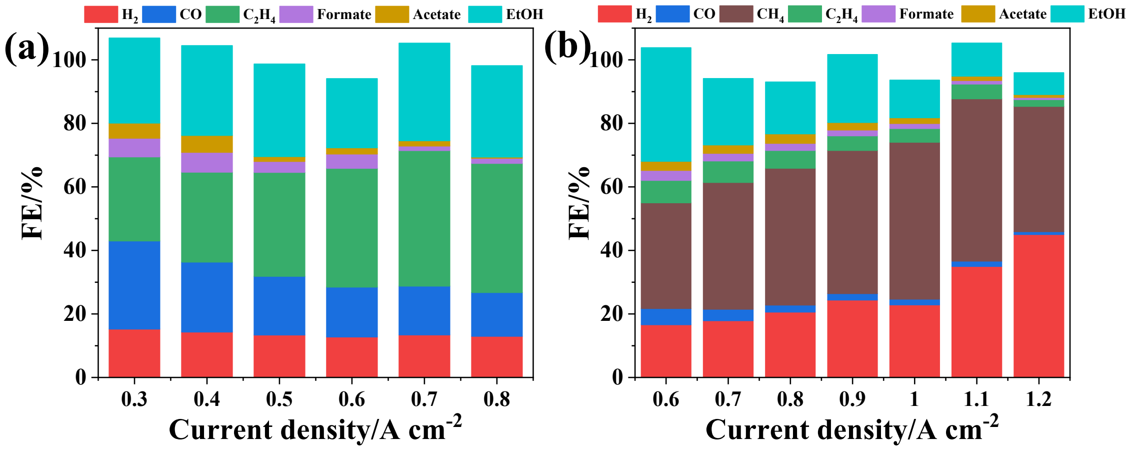

Figure S15. Faradaic efficiency for products with different applied current densities in the flow-cell configuration of (a) Cu_2O and (b) Cu_2O@RF.

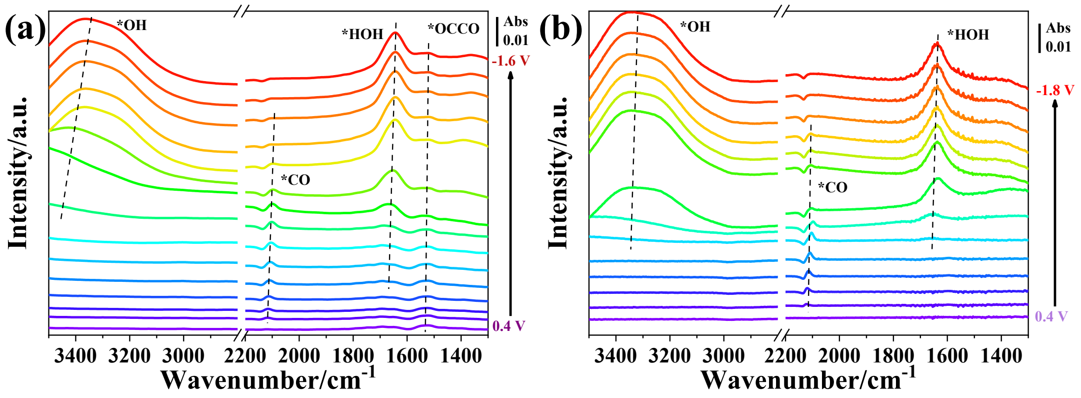

Figure S16. In-situ ATR-FTIR spectra of (a) Cu_2O and (b) Cu_2O@RF at various potentials in CO_2-saturated 0.1 M $KHCO_3$ H_2O solutions.

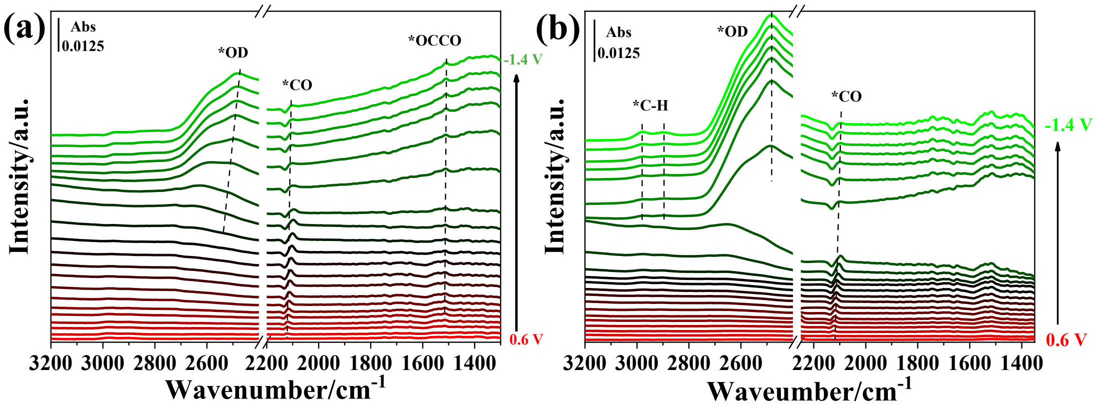

Figure S17. In-situ ATR-FTIR spectra of (a) Cu_2O and (b) Cu_2O@RF at potentials from 0.6 to -1.4 V versus RHE in CO_2-saturated 0.1 M $KHCO_3$ D_2O solutions.

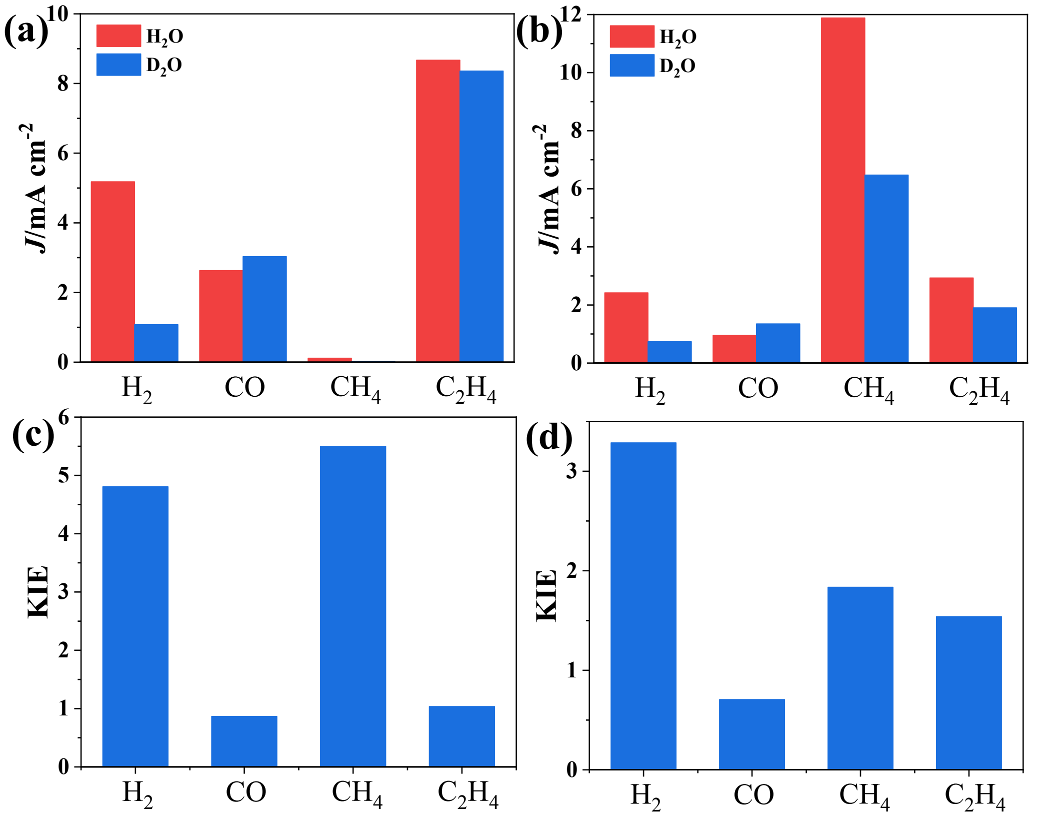

Figure S18. Partial current densities of different products at -1.8 V vs. RHE for (a) Cu_2O and (b) Cu_2O@RF; KIE of H/D to different products at -1.8 V vs. RHE for (c) Cu_2O and (d) Cu_2O@RF.

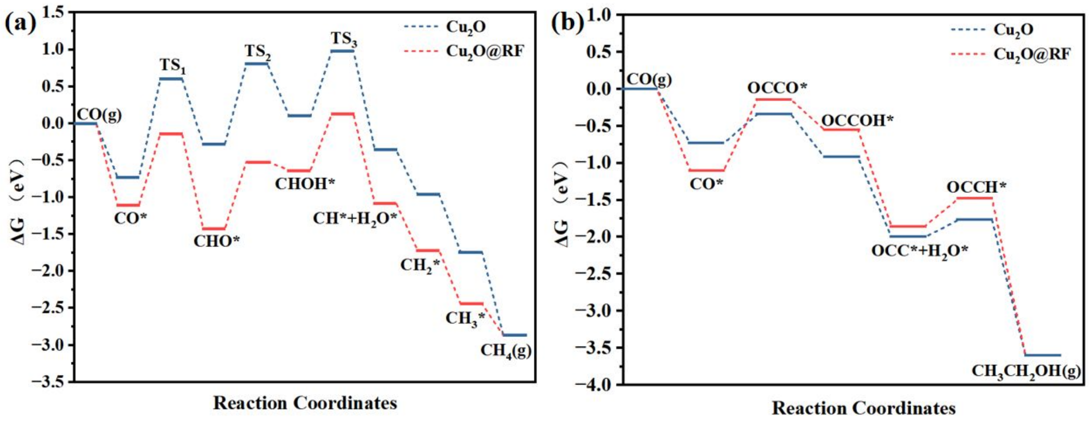

Figure S19. Gibbs free energy plots from CO to CH_4 and CH_3CH_2OH for Cu_2O (a) and Cu_2O@RF (b).

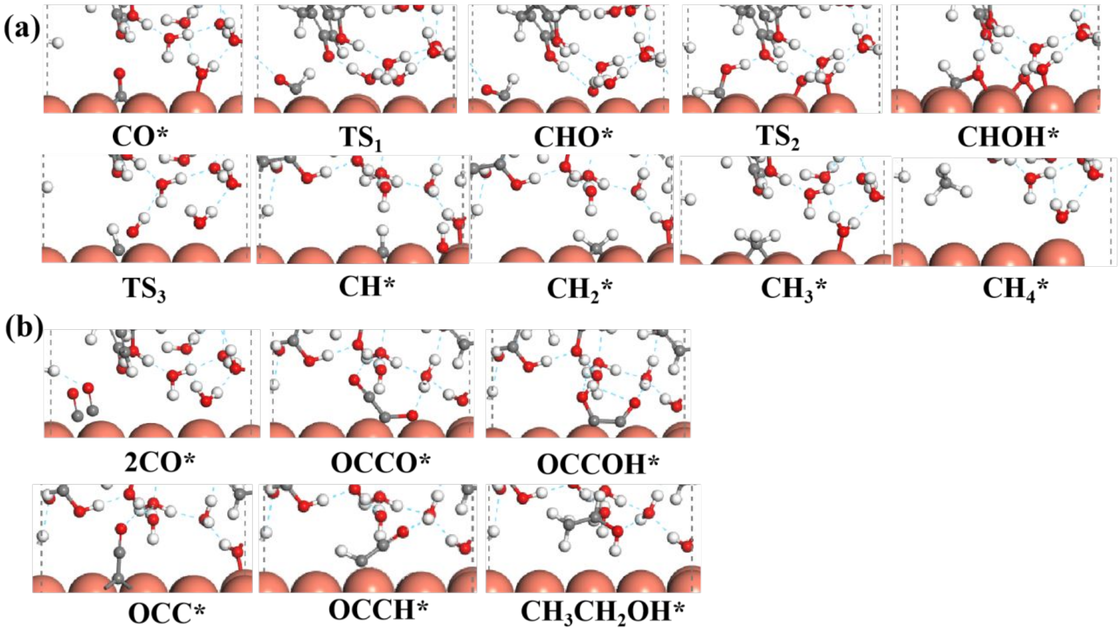

Figure S20. The optimization structures of Cu_2O@RF. The reaction mechanism includes CO to CH_4 (a) and CO to CH_3CH_2OH (b) .

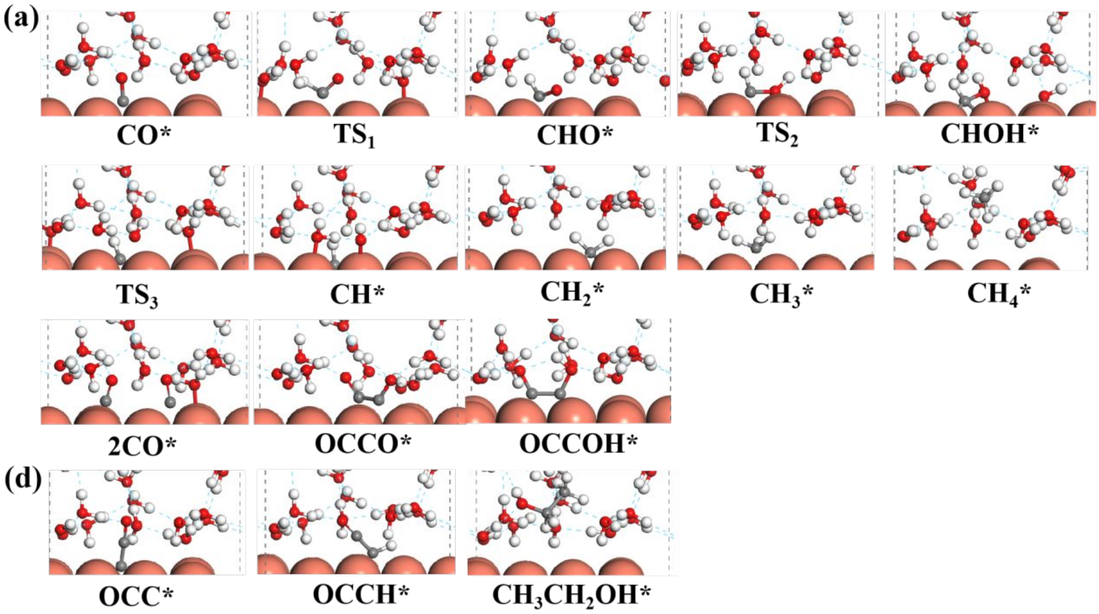

Figure S21. (a) The optimization structures of Cu_2O. The reaction mechanism includes CO to CH_4 (a) and CO to CH_3CH_2OH (b).

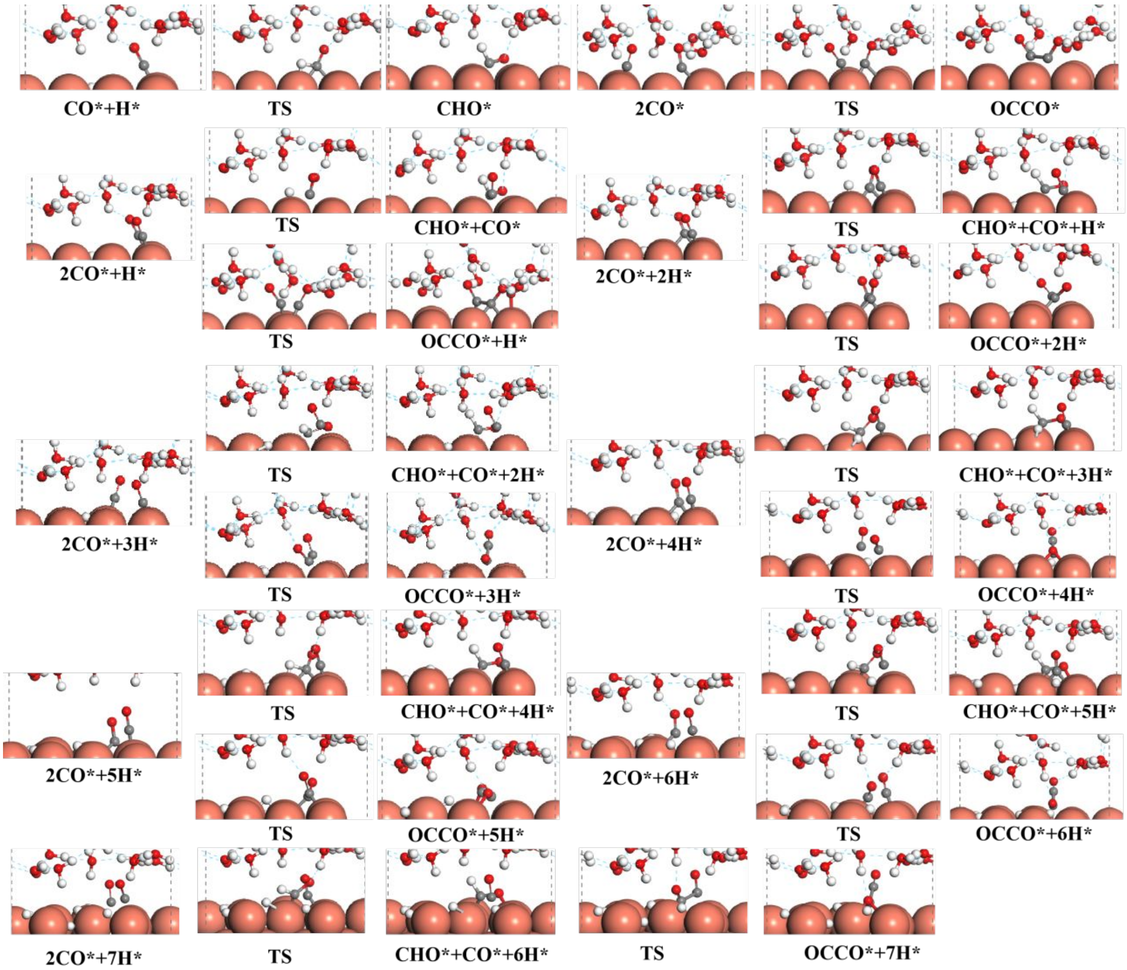

Figure S22. (a) The optimization structures of CHO* and OCCO at different coverage of H atom.

Table S1. Comparison of the CO_2 reduction reaction activity to methane over various catalysts in literature.

Catalyst	Current density/mA cm^{-2}	Faradaic efficiency/%	Reference
**Cu_2O@RF**	**1100**	**51.1**	**This work**
Cu-crown ether	600	51.2	7
Cu_1Sm_9–Ox	500	65	8
2Bn-Cu@UiO-67	420	70	9
Cu NNU-33	391	82	10
EOR-Cu(bipy)Br	300	51.2	11
Glutathione-modified copper	249.1	61.7	12
Ag@Cu_2O	240.5	74	13
Single-site copper	238	42	14
CoO/Cu/PTFE	225	60	15
Cu-PTFE	225	48	16
Low coordination number copper	219.4	62	17
La_2CuO_4 perovskite	207.8	56.3	18
Cu-DBC	203	80	19
Au-Cu	112	56	20
Single-atomic Cu-CeO_2	56	58	21
$Cu_{68}Ag_{32}$ nanowire	50	60	22
Cu@CuEu	45.4	74.7	23
Cu-CDs	40	78	24
$MoTe_2$ Layers	25.6	83	25
Copper clusters-C	22	81.7	26
Copper phosphate nanosheets	20	76	27
Copper phthalocyanine	19.7	66	28
Cu/ZnOx	18	36	29
Cu_2O/Cu	16	71	30
Cu_3Pd_xN	14.9	57.5	31
Cu_2O-CuHHTP	14.8	73	32
Copper nanowires	13.6	55	33
Cu_2O@Cu-MOF	13.3	63.2	34
Copper twin boundaries	11.9	59	35
Cu-DBC	11.4	56	36
CRD-Cu_3Pd	9.4	40.6	37
Cu NP-embedded carbon	9	45	38

HATNA-Cu-MOF	8.2	78	[39]
Cu@CuZn	7	52	[40]
Cu Nafion	1.8	88	[41]
Cu_2O/Cu@NC	1.5	23.9	[42]

References

(1) Nie, W.; Heim, G. P.; Watkins, N. B.; Agapie, T.; Peters, J. C. Organic Additive-derived Films on Cu Electrodes Promote Electrochemical CO_2 Reduction to C_{2+} Products Under Strongly Acidic Conditions. *Angewandte Chemie International Edition* **2023**, *62* (12), e202216102. DOI: 10.1002/anie.202216102.

(2) Kresse, G.; Furthmüller, J. Efficient iterative schemes for ab initio total-energy calculations using a plane-wave basis set. *Physical Review B* **1996**, *54* (16), 11169-11186. DOI: 10.1103/PhysRevB.54.11169. Kresse, G.; Hafner, J. Ab initio molecular-dynamics simulation of the liquid-metal--amorphous-semiconductor transition in germanium. *Physical Review B* **1994**, *49* (20), 14251-14269. DOI: 10.1103/PhysRevB.49.14251. Kresse, G.; Furthmüller, J. Efficiency of ab-initio total energy calculations for metals and semiconductors using a plane-wave basis set. *Computational Materials Science* **1996**, *6* (1), 15-50. DOI: 10.1016/0927-0256(96)00008-0. Perdew, J. P.; Burke, K.; Ernzerhof, M. Generalized Gradient Approximation Made Simple. *Physical Review Letters* **1996**, *77* (18), 3865-3868. DOI: 10.1103/PhysRevLett.77.3865.

(3) Henkelman, G.; Uberuaga, B. P.; Jónsson, H. A climbing image nudged elastic band method for finding saddle points and minimum energy paths. *The Journal of Chemical Physics* **2000**, *113* (22), 9901-9904. DOI: 10.1063/1.1329672.

(4) Lerch, D.; Klein, A.; Schmidt, A. K.; Müller, S.; Hammer, L.; Heinz, K.; Weinert, M. Unusual adsorption site of hydrogen on the unreconstructed Ir(100) surface. *Physical Review B* **2006**, *73*, 075430.

(5) Nørskov, J. K.; Rossmeisl, J.; Logadottir, A.; Lindqvist, L.; Kitchin, J. R.; Bligaard, T.; Jónsson, H. Origin of the Overpotential for Oxygen Reduction at a Fuel-Cell Cathode. *The Journal of Physical Chemistry B* **2004**, *108* (46), 17886-17892. DOI: 10.1021/jp047349j.

(6) Cheng, T.; Xiao, H.; Goddard, W. A., III. Reaction Mechanisms for the Electrochemical Reduction of CO_2 to CO and Formate on the Cu(100) Surface at 298 K from Quantum Mechanics Free Energy Calculations with Explicit Water. *Journal of the American Chemical Society* **2016**, *138* (42), 13802-13805. DOI: 10.1021/jacs.6b08534.

(7) Xu, K.; Li, J.; Liu, F.; Chen, X.; Zhao, T.; Cheng, F. Favoring CO Intermediate Stabilization and Protonation by Crown Ether for CO_2 Electromethanation in Acidic Media. *Angewandte Chemie International Edition* **2023**, *62* (50), e202311968. DOI: 10.1002/anie.202311968.

(8) Liu, J.; Li, P.; Bi, J.; Jia, S.; Wang, Y.; Kang, X.; Sun, X.; Zhu, Q.; Han, B. Switching between C_{2+} Products and CH_4 in CO_2 Electrolysis by Tuning the Composition and Structure of Rare-Earth/Copper Catalysts. *Journal of the American Chemical Society* **2023**, *145* (42), 23037-23047. DOI: 10.1021/jacs.3c05562.

(9) Chen, S.; Li, W.-H.; Jiang, W.; Yang, J.; Zhu, J.; Wang, L.; Ou, H.; Zhuang, Z.; Chen, M.; Sun, X.; et al. MOF Encapsulating N-Heterocyclic Carbene-Ligated Copper Single-Atom Site Catalyst towards Efficient Methane Electrosynthesis. *Angewandte Chemie International Edition* **2022**, *61* (4), e202114450. DOI: 10.1002/anie.202114450.

(10) Zhang, L.; Li, X.-X.; Lang, Z.-L.; Liu, Y.; Liu, J.; Yuan, L.; Lu, W.-Y.; Xia, Y.-S.; Dong, L.-Z.;

Yuan, D.-Q.; et al. Enhanced Cuprophilic Interactions in Crystalline Catalysts Facilitate the Highly Selective Electroreduction of CO_2 to CH_4. *Journal of the American Chemical Society* **2021**, *143* (10), 3808-3816. DOI: 10.1021/jacs.0c11450.

(11) Zhang, H.; Yang, Y.; Liang, Y.; Li, J.; Zhang, A.; Zheng, H.; Geng, Z.; Li, F.; Zeng, J. Molecular Stabilization of Sub-Nanometer Cu Clusters for Selective CO_2 Electromethanation. *ChemSusChem* **2022**, *15* (1), e202102010. DOI: 10.1002/cssc.202102010.

(12) Shi, Y.; Sun, K.; Shan, J.; Li, H.; Gao, J.; Chen, Z.; Sun, C.; Shuai, Y.; Wang, Z. Selective CO_2 Electromethanation on Surface-Modified Cu Catalyst by Local Microenvironment Modulation. *ACS Catalysis* **2022**, *12* (14), 8252-8258. DOI: 10.1021/acscatal.2c01544.

(13) Xiong, L.; Zhang, X.; Chen, L.; Deng, Z.; Han, S.; Chen, Y.; Zhong, J.; Sun, H.; Lian, Y.; Yang, B.; et al. Geometric Modulation of Local CO Flux in Ag@Cu_2O Nanoreactors for Steering the CO_2RR Pathway toward High-Efficacy Methane Production. *Advanced Materials* **2021**, *33* (32), 2101741. DOI: 10.1002/adma.202101741.

(14) Zhang, T.; Verma, S.; Kim, S.; Fister, T. T.; Kenis, P. J. A.; Gewirth, A. A. Highly dispersed, single-site copper catalysts for the electroreduction of CO_2 to methane. *Journal of Electroanalytical Chemistry* **2020**, *875*, 113862. DOI: 10.1016/j.jelechem.2020.113862.

(15) Li, Y.; Xu, A.; Lum, Y.; Wang, X.; Hung, S.-F.; Chen, B.; Wang, Z.; Xu, Y.; Li, F.; Abed, J.; et al. Promoting CO_2 methanation via ligand-stabilized metal oxide clusters as hydrogen-donating motifs. *Nature Communications* **2020**, *11* (1), 6190. DOI: 10.1038/s41467-020-20004-7.

(16) Wang, X.; Xu, A.; Li, F.; Hung, S.-F.; Nam, D.-H.; Gabardo, C. M.; Wang, Z.; Xu, Y.; Ozden, A.; Rasouli, A. S.; et al. Efficient Methane Electrosynthesis Enabled by Tuning Local CO_2 Availability. *Journal of the American Chemical Society* **2020**, *142* (7), 3525-3531. DOI: 10.1021/jacs.9b12445.

(17) Xu, Y.; Li, F.; Xu, A.; Edwards, J. P.; Hung, S.-F.; Gabardo, C. M.; O'Brien, C. P.; Liu, S.; Wang, X.; Li, Y.; et al. Low coordination number copper catalysts for electrochemical CO_2 methanation in a membrane electrode assembly. *Nature Communications* **2021**, *12* (1), 2932. DOI: 10.1038/s41467-021-23065-4.

(18) Chen, S.; Su, Y.; Deng, P.; Qi, R.; Zhu, J.; Chen, J.; Wang, Z.; Zhou, L.; Guo, X.; Xia, B. Y. Highly Selective Carbon Dioxide Electroreduction on Structure-Evolved Copper Perovskite Oxide toward Methane Production. *ACS Catalysis* **2020**, *10* (8), 4640-4646. DOI: 10.1021/acscatal.0c00847.

(19) Zhang, Y.; Dong, L.-Z.; Li, S.; Huang, X.; Chang, J.-N.; Wang, J.-H.; Zhou, J.; Li, S.-L.; Lan, Y.-Q. Coordination environment dependent selectivity of single-site-Cu enriched crystalline porous catalysts in CO_2 reduction to CH_4. *Nature Communications* **2021**, *12* (1), 6390. DOI: 10.1038/s41467-021-26724-8.

(20) Wang, X.; Ou, P.; Wicks, J.; Xie, Y.; Wang, Y.; Li, J.; Tam, J.; Ren, D.; Howe, J. Y.; Wang, Z.; et al. Gold-in-copper at low *CO coverage enables efficient electromethanation of CO_2. *Nature Communications* **2021**, *12* (1), 3387. DOI: 10.1038/s41467-021-23699-4.

(21) Wang, Y.; Chen, Z.; Han, P.; Du, Y.; Gu, Z.; Xu, X.; Zheng, G. Single-Atomic Cu with Multiple Oxygen Vacancies on Ceria for Electrocatalytic CO_2 Reduction to CH_4. *ACS Catalysis* **2018**, *8* (8), 7113-

7119. DOI: 10.1021/acscatal.8b01014.
(22) Chang, C.-J.; Lin, S.-C.; Chen, H.-C.; Wang, J.; Zheng, K. J.; Zhu, Y.; Chen, H. M. Dynamic Reoxidation/Reduction-Driven Atomic Interdiffusion for Highly Selective CO_2 Reduction toward Methane. *Journal of the American Chemical Society* **2020**, *142* (28), 12119-12132. DOI: 10.1021/jacs.0c01859.
(23) Shan, J.; Sun, K.; Li, H.; Xu, P.; Sun, J.; Wang, Z. Composition regulation and defects introduction via amorphous CuEu alloy shell for efficient CO_2 electroreduction toward methane. *Journal of CO2 Utilization* **2020**, *41*, 101285. DOI: 10.1016/j.jcou.2020.101285.
(24) Cai, Y.; Fu, J.; Zhou, Y.; Chang, Y.-C.; Min, Q.; Zhu, J.-J.; Lin, Y.; Zhu, W. Insights on forming N,O-coordinated Cu single-atom catalysts for electrochemical reduction CO_2 to methane. *Nature Communications* **2021**, *12* (1), 586. DOI: 10.1038/s41467-020-20769-x.
(25) Liu, X.; Yang, H.; He, J.; Liu, H.; Song, L.; Li, L.; Luo, J. Highly Active, Durable Ultrathin $MoTe_2$ Layers for the Electroreduction of CO_2 to CH_4. *Small* **2018**, *14* (16), 1704049. DOI: 10.1002/smll.201704049.
(26) Hu, Q.; Han, Z.; Wang, X.; Li, G.; Wang, Z.; Huang, X.; Yang, H.; Ren, X.; Zhang, Q.; Liu, J.; et al. Facile Synthesis of Sub-Nanometric Copper Clusters by Double Confinement Enables Selective Reduction of Carbon Dioxide to Methane. *Angewandte Chemie International Edition* **2020**, *59* (43), 19054-19059. DOI: 10.1002/anie.202009277.
(27) Zhang, B.; Zhang, J.; An, P.; Su, Z.; Wan, Q.; Tan, X.; Zheng, L. Steering CO_2 electroreduction toward methane or ethylene production. *Nano Energy* **2021**, *88*, 106239. DOI: 10.1016/j.nanoen.2021.106239.
(28) Weng, Z.; Wu, Y.; Wang, M.; Jiang, J.; Yang, K.; Huo, S.; Wang, X.-F.; Ma, Q.; Brudvig, G. W.; Batista, V. S.; et al. Active sites of copper-complex catalytic materials for electrochemical carbon dioxide reduction. *Nature Communications* **2018**, *9* (1), 415. DOI: 10.1038/s41467-018-02819-7.
(29) Wang, R.; Jiang, R.; Dong, C.; Tong, T.; Li, Z.; Liu, H.; Du, X.-W. Engineering a Cu/ZnOx Interface for High Methane Selectivity in CO_2 Electrochemical Reduction. *Industrial & Engineering Chemistry Research* **2021**, *60* (1), 273-280. DOI: 10.1021/acs.iecr.0c04718.
(30) Deng, B.; Huang, M.; Li, K.; Zhao, X.; Geng, Q.; Chen, S.; Xie, H.; Dong, X. a.; Wang, H.; Dong, F. The Crystal Plane is not the Key Factor for CO_2-to-Methane Electrosynthesis on Reconstructed Cu_2O Microparticles. *Angewandte Chemie International Edition* **2022**, *61* (7), e202114080. DOI: 10.1002/anie.202114080.
(31) Li, T.; Wang, J.; Zhu, S.; Delmo, E. P.; Sun, F.; Zhang, L.; Gu, M.; Shao, M. Cu_3PdxN nanocrystals for efficient CO_2 electrochemical reduction to methane. *Electrochimica Acta* **2021**, *371*, 137793. DOI: 10.1016/j.electacta.2021.137793.
(32) Yi, J.-D.; Xie, R.; Xie, Z.-L.; Chai, G.-L.; Liu, T.-F.; Chen, R.-P.; Huang, Y.-B.; Cao, R. Highly Selective CO_2 Electroreduction to CH_4 by In Situ Generated Cu_2O Single-Type Sites on a Conductive MOF: Stabilizing Key Intermediates with Hydrogen Bonding. *Angewandte Chemie International Edition* **2020**, *59* (52), 23641-23648. DOI: 10.1002/anie.202010601.

(33) Li, Y.; Cui, F.; Ross, M. B.; Kim, D.; Sun, Y.; Yang, P. Structure-Sensitive CO_2 Electroreduction to Hydrocarbons on Ultrathin 5-fold Twinned Copper Nanowires. *Nano Letters* **2017**, *17* (2), 1312-1317. DOI: 10.1021/acs.nanolett.6b05287.
(34) Tan, X.; Yu, C.; Zhao, C.; Huang, H.; Yao, X.; Han, X.; Guo, W.; Cui, S.; Huang, H.; Qiu, J. Restructuring of Cu_2O to Cu_2O@Cu-Metal–Organic Frameworks for Selective Electrochemical Reduction of CO_2. *ACS Applied Materials & Interfaces* **2019**, *11* (10), 9904-9910. DOI: 10.1021/acsami.8b19111.
(35) Tang, C.; Shi, J.; Bai, X.; Hu, A.; Xuan, N.; Yue, Y.; Ye, T.; Liu, B.; Li, P.; Zhuang, P.; et al. CO_2 Reduction on Copper's Twin Boundary. *ACS Catalysis* **2020**, *10* (3), 2026-2032. DOI: 10.1021/acscatal.9b03814.
(36) Liu, Y.-Y.; Zhu, H.-L.; Zhao, Z.-H.; Huang, N.-Y.; Liao, P.-Q.; Chen, X.-M. Insight into the Effect of the d-Orbital Energy of Copper Ions in Metal–Organic Frameworks on the Selectivity of Electroreduction of CO_2 to CH_4. *ACS Catalysis* **2022**, *12* (5), 2749-2755. DOI: 10.1021/acscatal.1c04805.
(37) Zhu, W.; Zhang, L.; Yang, P.; Chang, X.; Dong, H.; Li, A.; Hu, C.; Huang, Z.; Zhao, Z.-J.; Gong, J. Morphological and Compositional Design of Pd–Cu Bimetallic Nanocatalysts with Controllable Product Selectivity toward CO_2 Electroreduction. *Small* **2018**, *14* (7), 1703314. DOI: 10.1002/smll.201703314.
(38) Li, J.; Chen, W.; Wang, M.; Zhu, H. Self-Supporting Copper-Based Electrode by Electrospinning for Reduction of Carbon Dioxide to Methane. *Energy Technology* **2021**, *9* (12), 2100714. DOI: 10.1002/ente.202100714.
(39) Liu, Y.; Li, S.; Dai, L.; Li, J.; Lv, J.; Zhu, Z.; Yin, A.; Li, P.; Wang, B. The Synthesis of Hexaazatrinaphthylene-Based 2D Conjugated Copper Metal-Organic Framework for Highly Selective and Stable Electroreduction of CO_2 to Methane. *Angewandte Chemie International Edition* **2021**, *60* (30), 16409-16415. DOI: 10.1002/anie.202105966.
(40) Varandili, S. B.; Stoian, D.; Vavra, J.; Rossi, K.; Pankhurst, J. R.; Guntern, Y. T.; López, N.; Buonsanti, R. Elucidating the structure-dependent selectivity of CuZn towards methane and ethanol in CO_2 electroreduction using tailored Cu/ZnO precatalysts. *Chemical Science* **2021**, *12* (43), 14484-14493, 10.1039/D1SC04271H. DOI: 10.1039/D1SC04271H.
(41) Pan, H.; Barile, C. J. Electrochemical CO_2 reduction to methane with remarkably high Faradaic efficiency in the presence of a proton permeable membrane. *Energy & Environmental Science* **2020**, *13* (10), 3567-3578, 10.1039/D0EE02189J. DOI: 10.1039/D0EE02189J.
(42) Huidong Jin, L. X., Xiang Zhang, Yuebin Lian, Si Chen, Yongtao Lu, Zhao Deng, Yang Peng. Cu-Based Catalyst Derived from Nitrogen-Containing Metal Organic Frameworks for Electroreduction of CO_2. *Acta Phys. -Chim. Sin.* **2021**, *37* (11), 2006017. DOI: 10.3866/pku.whxb202006017.
